# Supplementary material for: Coadaptation fostered by the SLIT2-ROBO1 axis facilitates liver metastasis of pancreatic ductal adenocarcinoma
Source: Nat Commun. 2023 Feb 15;14:861. doi: 10.1038/s41467-023-36521-0 (PMC9932171; doi:10.1038/s41467-023-36521-0)
Supplement: Supplementary file 1 — Supplementary Information [file 41467_2023_36521_MOESM1_ESM.pdf]

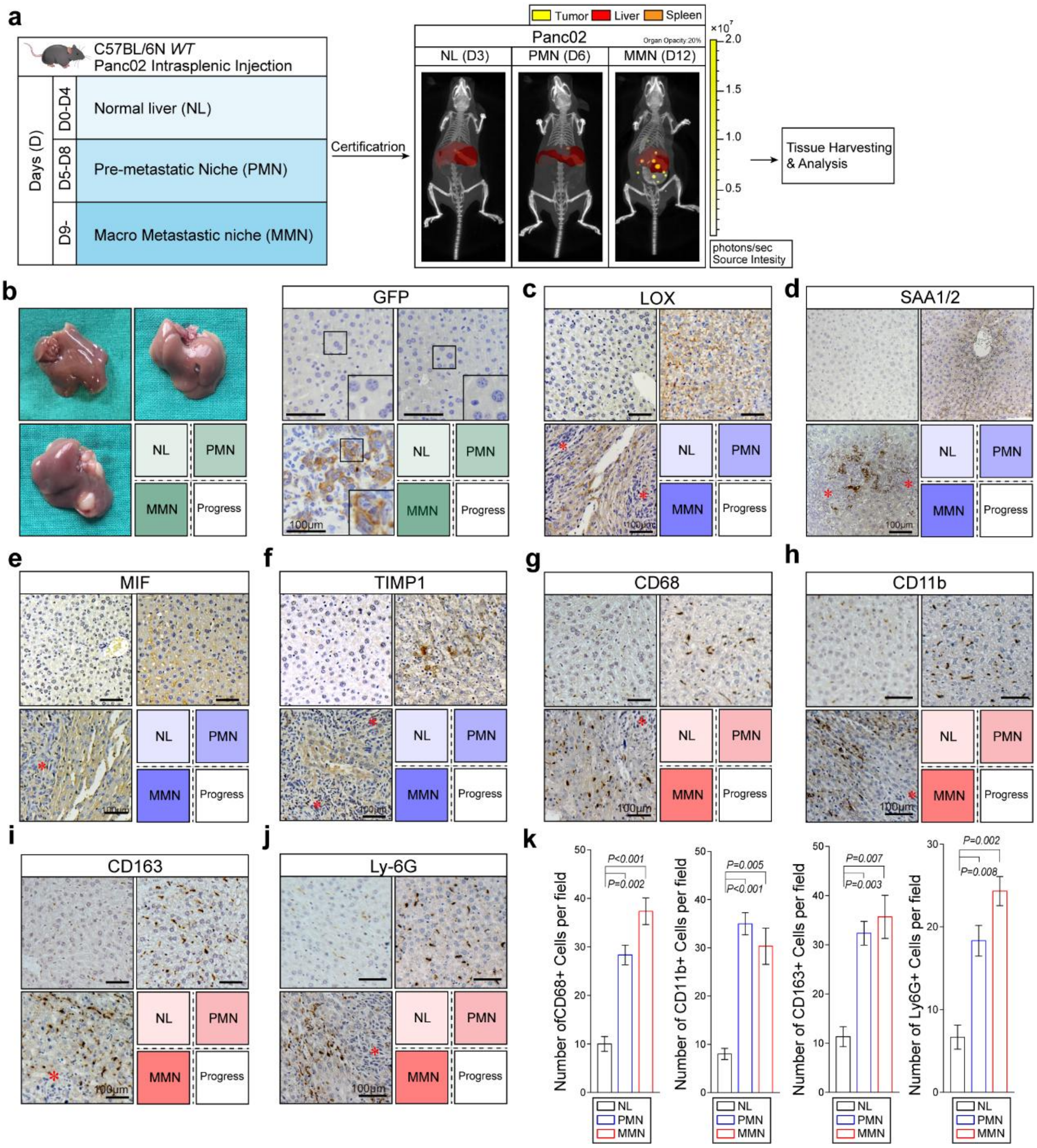


**Supplementary Figure 1. Detection of Premetastatic niche in Panc02 injected mouse model**

(**a**) Liver metastasis model established by intrasplenic injection of murine Panc02$^{luc}$ cell line followed by CT combined 3D organ reconstruction bioluminescence imaging assessing the time point of metastasis. Mice experienced intrasplenic injection photographed on day 3 (D3), day 6 (D6) and day12 (D12). Red, reconstructed liver; orange, reconstructed spleen; yellow, signals of tumour niches. Scale colour bar: $2.00\times10^5$-$2.00\times10^7$. (n=5 mice per group). (**b**) Representative livers of liver metastasis mouse model on progress Normal liver (NL) (D3), Premetastatic niche (PMN) (D6) and Macrometastatic niche (MMN) (D12) after injection (n=5 mice per group) and

respective IHC-P staining of GFP on left lobule of model (n=5 samples per group, 3 fields assessed per sample). Scale bars, 100μm. (**c-f**) Representative IHC-P staining of PMN specific secreted protein markers: LOX (**c**), SAA1/2 (**d**), MIF (**e**) or TIMP1 (**f**) on left lobules of livers of mice model on progress NL (D3), PMN (D6) and MMN (D12) (n=5 samples per group, 3 fields assessed per sample). Scale bars, 100μm. * represented metastatic niches. (**g-k**) Representative IHC-P staining of PMN residing Tumour associated macrophages (TAMs) markers: CD68 (**g**), CD11b (**h**), CD163 (**i**) or Ly-6G (**j**) and cell counting of which (**k**) in left lobules of livers of mice model on progress NL (D3), PMN (D6) and MMN (D12) n=5 samples per group, 3 fields assessed per sample). Scale bars, 100μm. * represented metastatic niches. (n=5 samples per group, 3 fields assessed per sample, mean±s.e.m., two tailed unpaired *t*-test). Source data are provided as Source Data file.

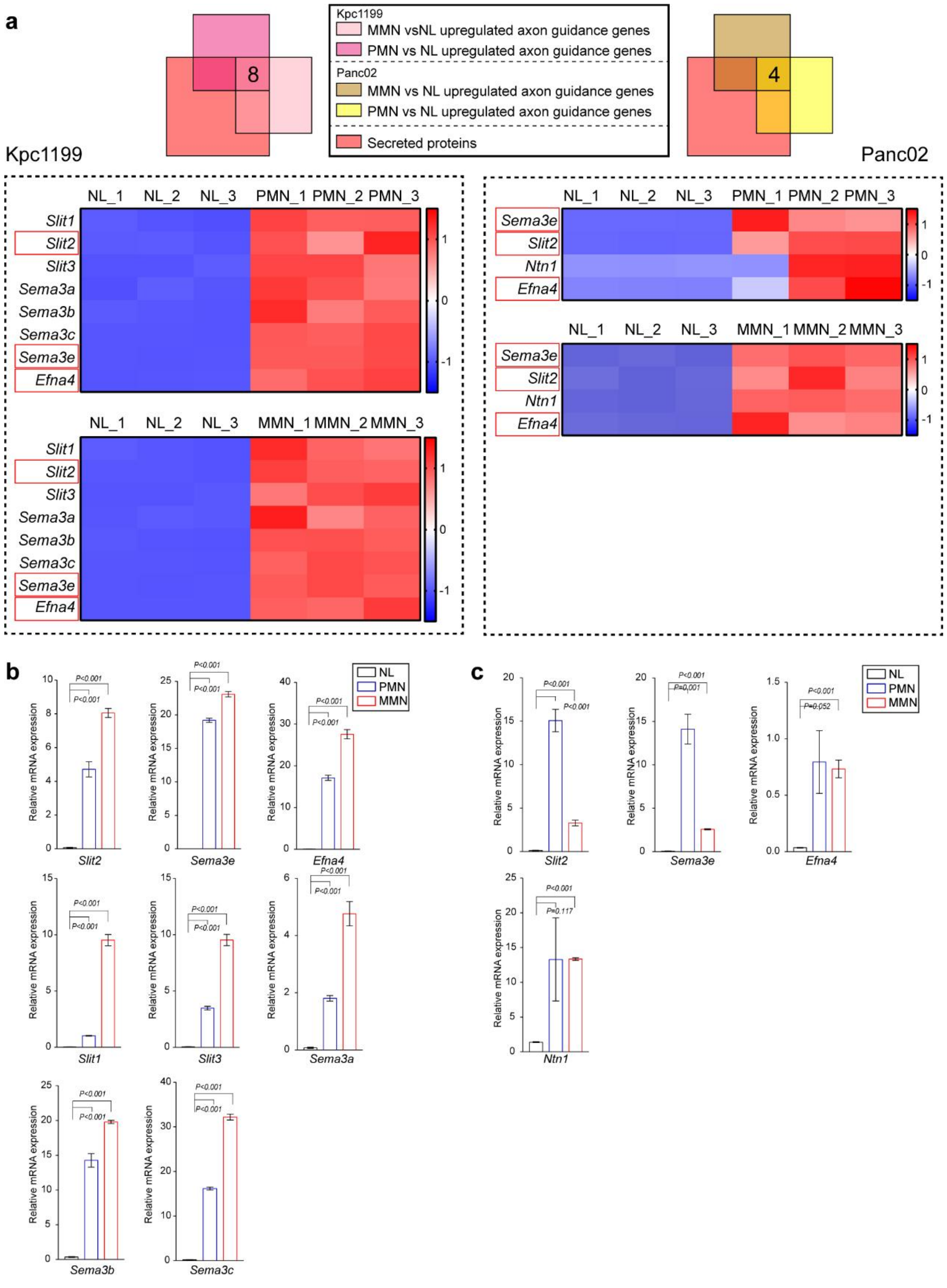


**Supplementary Figure 2. Secreted proteins of Axon Guidance pathway are upregulated at Premetastatic niche and Macrometastatic niche**

(**a**) Heatmap displaying the upregulated secreted axon guidance genes in Premetastatic niche (PMN) and Macrometastatic niche (MMN) of Kpc1199 (left) and Panc02 (right) treated mouse models and the overlapped ones are recognized. padj <

0.01 | log2FoldChange |> 3. (**b-c**) Shown is the relative mRNA expression of upregulated secreted axon guidance genes in Kpc1199 (**b**) or Panc02 (**c**) treated mouse models respectively. (n=3 samples per group, mean±s.e.m., two tailed unpaired *t*-test). Source data are provided as Source Data file.

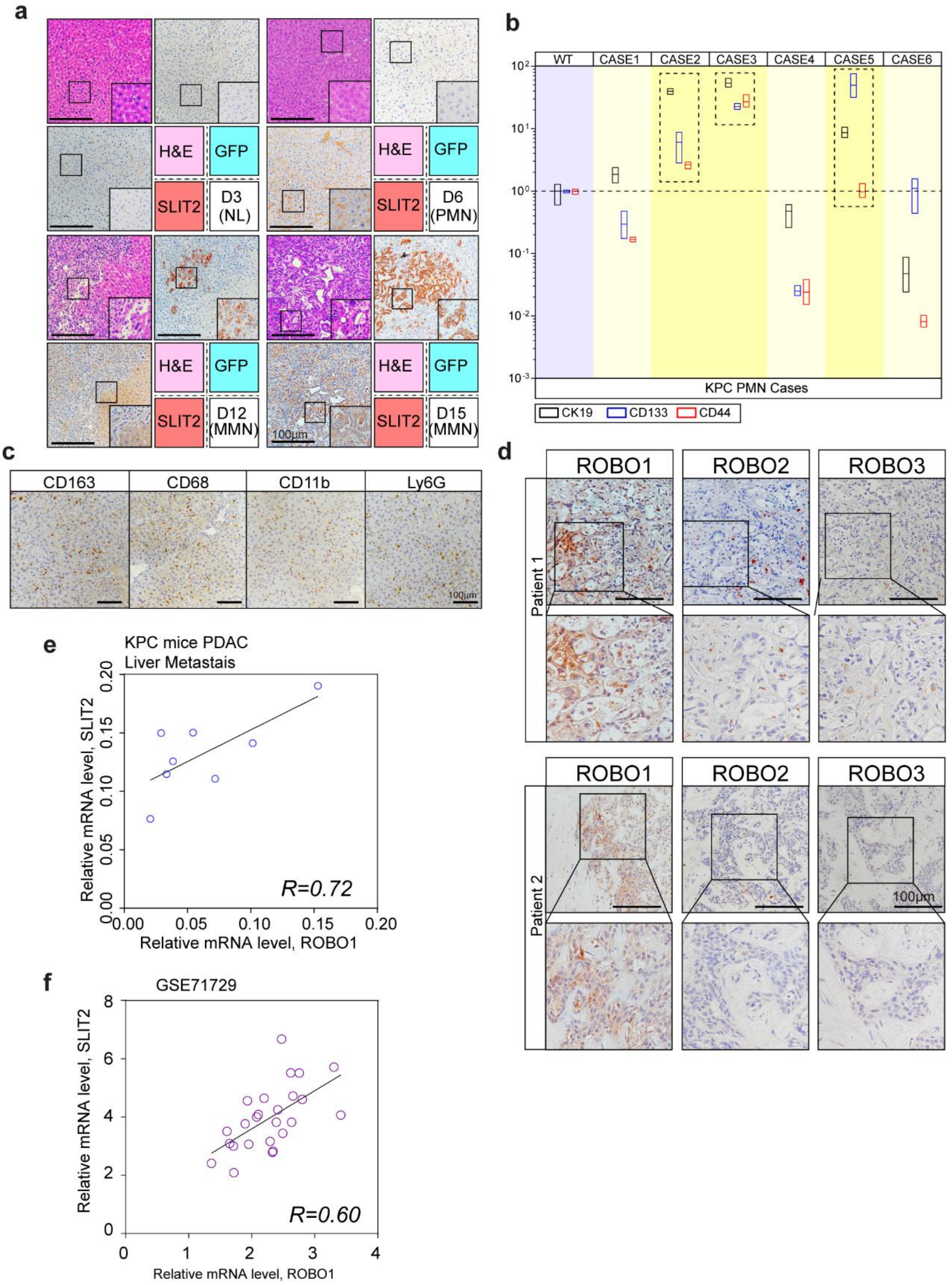


**Supplementary Figure 3. SLIT2 and ROBO family expressed at liver metastasis**

(**a**) Representative IHC-P staining of H&E, GFP or SLIT2 in left lobule of livers of Panc02 injected mouse models on Normal liver (NL) (D3), Premetastatic niche (PMN) (D6) and Macrometastatic niche (MMN) (D12 and D15) (n=5 samples per group, 3 fields assessed per sample). Scale bars, 100μm. (**b**) Real-time PCR measuring the

expressions of Disseminated tumour cells (DTCs) markers in PDAC: CK 19, CD133 or CD44 in left hepatic lobules of 6 case *Kras*$^{G12D/+}$/*Trp53*$^{R172H/+}$/*Pdx1*-Cre (KPC) mouse model. Up-regulation of either 2 of the 3 markers was recognized as DTCs positive (n=6 mice, n=3 technical repeats per sample). (**c**) Representative IHC-P staining of PMN residing macrophages markers CD163, CD68, CD11b and Ly-6G in left lobule of livers of DTCs detected KPC mice (n=6 samples per group, 3 fields assessed per sample). Scale bars, 100μm. (**d**) Representative IHC-P staining of ROBO family (ROBO1, ROBO2 and ROBO3) on serial sections of liver metastatic niches of PDAC patients (n=35 cases, 3 fields assessed per sample). Scale bars, 100μm. (**e**) Correlation analysis of ROBO1 and SLIT2 mRNA levels in liver metastasis in the KPC spontaneous PDAC liver metastasis model (n=8 mice). (**f**) Correlation analysis of ROBO1 and SLIT2 mRNA levels in liver metastasis of patients in GSE71729. Source data are provided as Source Data file.

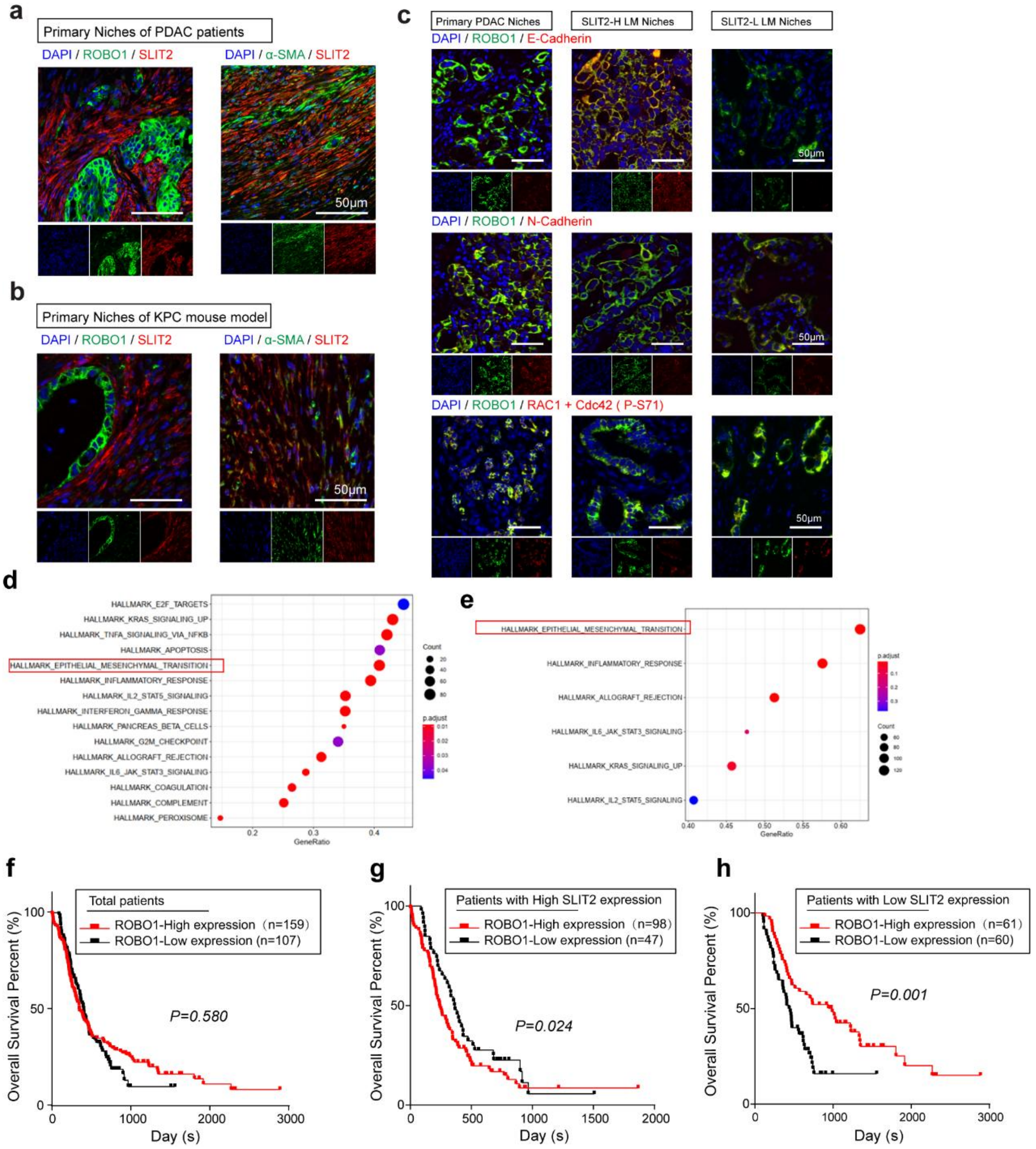


**Supplementary Figure 4. SLIT2 and ROBO1 expressions in primary PDAC niches and prognosis**

(**a-b**) Representative IF staining showing the location of SLIT2 and ROBO1 or α-SMA in primary niches of PDAC patients (**a**) and *Kras*$^{G12D/+}$/*Trp53*$^{R172H/+}$/*Pdx1*-Cre (KPC) mouse model. SLIT2, red; ROBO1 or α-SMA, green; DAPI, blue (n=10 patients, 3 fields assessed per sample; n=5 mice, 3 fields assessed per sample). Scale

bar, 50 μm.

(**c**) Representative IF staining showing ROBO1 expression with E-cadherin, N-cadherin or Cdc42 expressions in primary tumour niches and liver metastatic (LM) niches with high or low SLIT2 expression of PDAC patients. ROBO1, green; E-cadherin, N-cadherin or Cdc42, red; DAPI, blue (n=10 patients, 3 fields assessed per sample) Scale bar, 50 μm. (**d-e**) Gene set enrichment analysis (GSEA) based on the gene expression of ROBO1 in GSE15471 (n=19 per group) (**d**) and TCGA PAAD database (n=60 per group) (Wilcox test. FDR<0.05 and logFC>1) (**e**). (**f-h**) Kaplan–Meier analysis evaluating the relationship between ROBO1 expression and prognosis in PDAC patients with high SLIT2 expression (**g**) or low SLIT2 expression (**h**) according to IHC-P staining scores of primary tumours (n=266 cases). Source data are provided as Source Data file.

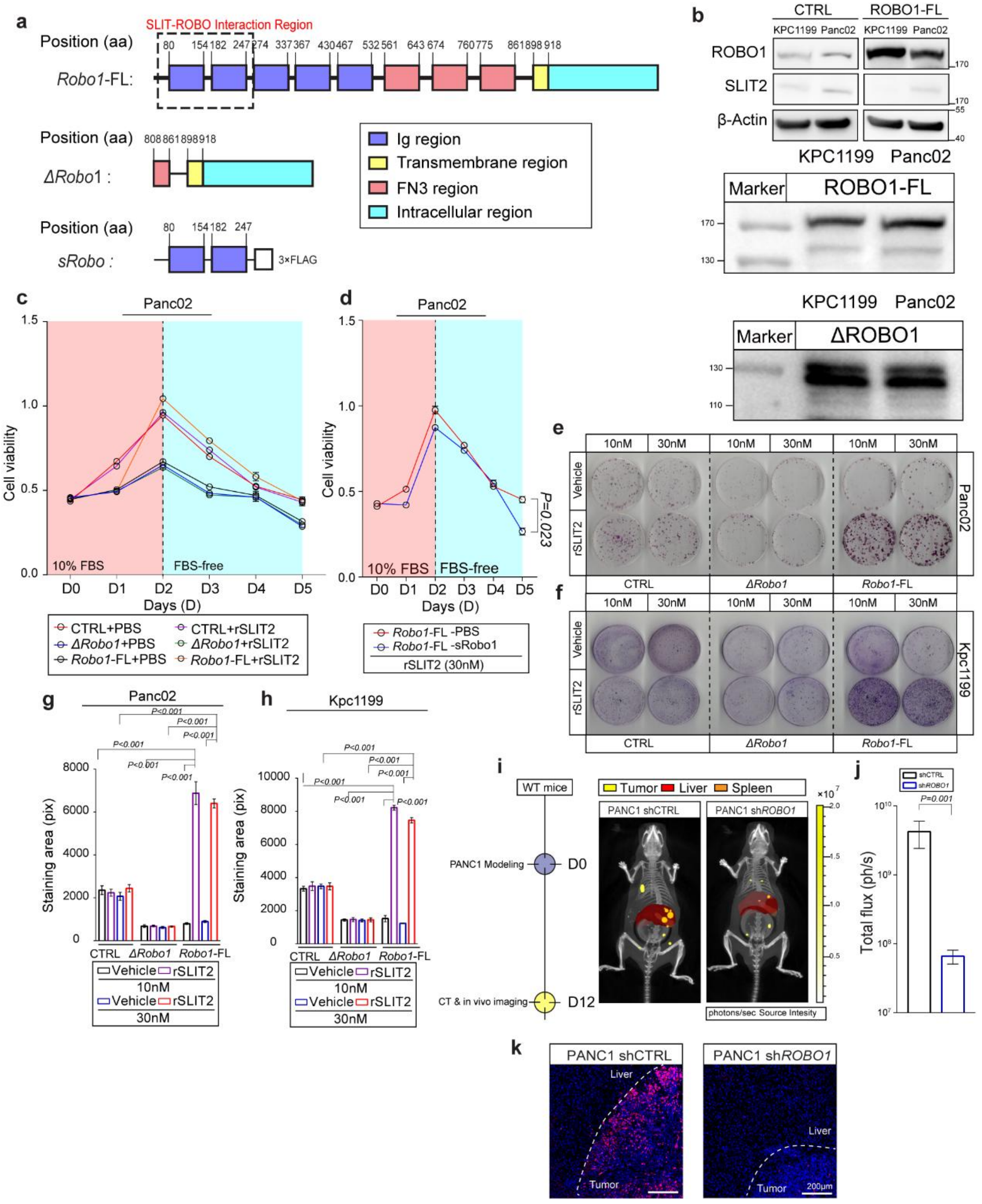


**Supplementary Figure 5. Characterization of ROBO1 molecular**

(**a**) Shown is the sequences and structures of *Robo1*-FL, *ΔRobo1* or *sRobo*. (**b**) WB displaying the expression level of ROBO1 and molecular weights of ROBO1-FL or ΔROBO1(n=3 technical repeats per test). (**c**) Cell viability of Panc02$^{CTRL}$,

Panc02$^{\Delta ROBO1}$ and Panc02$^{ROBO1\text{-FL}}$ with (+rSLIT2) or without (+PBS) 30nM rSLIT2 exposure, P values are as follow: CTRL+PBS vs *ΔROBO1*+PBS: *P<0.001*, CTRL+PBS vs *ROBO1*-FL+PBS: *P<0.001*, *ΔROBO1*+PBS vs *ROBO1*-FL+PBS: *P=0.103*, CTRL+rSLIT2 vs *ΔROBO1*+rSLIT2: *P<0.001*, CTRL+rSLIT2 vs *ROBO1*-FL+rSLIT2: *P=0.212*, *ΔROBO1*+rSLIT2 vs *ROBO1*-FL+rSLIT2 *P<0.001*, *ΔROBO1*+PBS vs *ΔROBO1*+rSLIT2: *P=0.700*, *ROBO1*-FL+PBS vs *ROBO1*-FL+rSLIT2: *P<0.001* (n=2 biological replicates, mean±s.e.m. Repeat measures ANOVA). Y-axis represented OD values at 450nm. (**d**) Cell viability of SW-1990$^{ROBO1\text{-FL}}$ exposed with 30nM rSLIT2 with or without sROBO treatment (n=2 biological replicates, mean±s.e.m. Repeat measures ANOVA). Y-axis represented OD values at 450nm. (**e-h**) Colony formation assay evaluating outgrowth ability of Panc02 (**e, g**) or Kpc1199 (**f, h**) expressed ROBO1-FL or ΔROBO1 exposed to 10nM or 30nM rSLIT2 (n=2 biological replicates, mean±s.e.m.; two tailed unpaired *t*-test); (**i-j**) Representative CT combined with 3D organ reconstruction bioluminescence imaging displaying PANC1$^{\text{shCTRL}}$ and PANC1$^{\text{Sh}ROBO1}$ cell-injected liver metastasis mouse models (n=5 mice per group, mean±s.e.m.; two-tailed unpaired *t* test). Scale colour bar: $2.00\times10^{5}$-$2.00\times10^{7}$. (**k**) Representative staining of Ki67 in liver metastatic niches of PANC1$^{\text{shCTRL}}$ and PANC1$^{\text{Sh}ROBO1}$ modelled mice (n=3 technical repeats per test, 3 fields assessed per sample). Ki67, red; DAPI, blue. Scale bars, 200μm. Source data are provided as Source Data file.

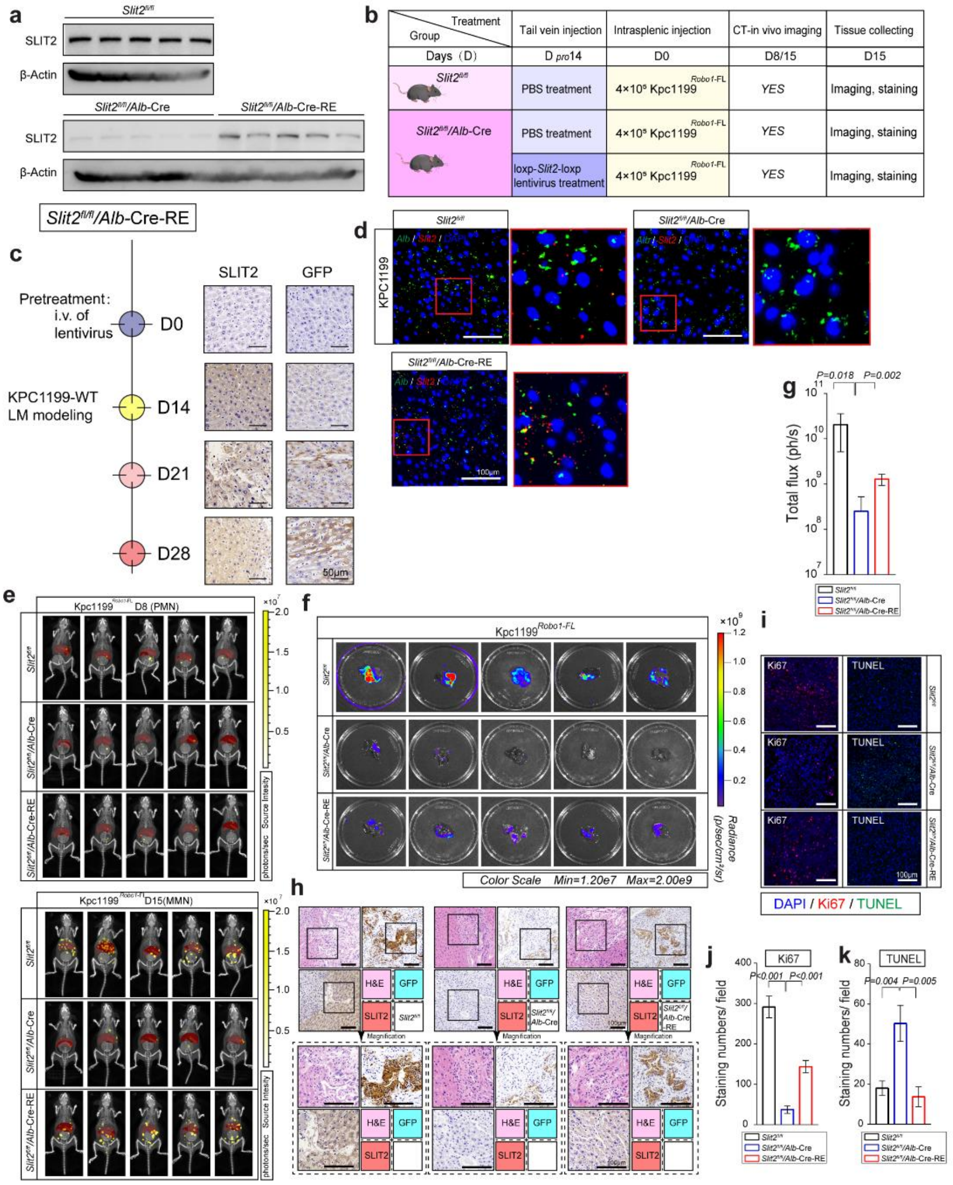


**Supplementary Figure 6. SLIT2 promotes outgrowth of ROBO1$^{+}$ tumour cells at liver metastasis *in vivo* (I)**

(**a**) WB assessing the expressions of SLIT2 in Kpc1199$^{Robo1\text{-FL}}$ stimulated livers of CTRL, *Slit2*/CKO or *Slit2*/CKO-RE intrasplenic injection models. (**b**) Shown is brief procedure of animal experiment involving CTRL, *Slit2*/CKO or *Slit2*/CKO-RE mice. (**c**) Representative IHC-P staining of SLIT2 and GFP on *Slit2*/CKO-RE mice at D0,

D14, D21 and D28 after modelled by Kpc1199 cell line respectively. (n=5 mice per group, 3 fields assessed per sample). Scale bars, 50 µm. (**d**) Representative RNAscope staining examining *Slit2* and *Alb* mRNA in livers of Kpc1199 modelled $Slit2^{fl/fl}$ or $Slit2^{fl/fl}$/*Alb*-Cre mice with or without Lenti-*loxp-Slit2* injection. *Alb*, green; *Slit2*, red; DAPI, blue. (n=5 samples, 3 fields assessed per sample). Scale bar, 100 µm. (**e**) CT combined with 3D organ reconstruction bioluminescence imaging assessing liver metastasis of $Kpc1199^{Robo1\text{-FL}}$ intrasplenically injected CTRL, *Slit2*/CKO or *Slit2*/CKO-RE mice on PMN (upper) or MMN (lower) (n=5 mice per group). Scale colour bars: $2.00\times10^{5}$-$2.00\times10^{7}$. Red, reconstructed liver; orange, reconstructed spleen; yellow, signals of tumour niches. (**f-g**) Assessment of separated metastasized livers from $Kpc1199^{Robo1\text{-FL}}$ modelled CTRL, *Slit2*/CKO or *Slit2*/CKO-RE mice (n=5 mice per group, mean±s.e.m.; two tailed unpaired *t*-test). Scale colour bar: $1.20\times10^{7}$-$2.00\times10^{9}$. (**h**) Representative IHC-P staining of H&E, GFP or SLIT2 in left lobule of livers of $Kpc1199^{Robo1\text{-FL}}$ modelled CTRL, *Slit2*/CKO or *Slit2*/CKO-RE mice (n=5 samples per group, 3 fields assessed per sample). Scale bars, 100µm. (**i-k**) Representative staining of Ki67 and TUNEL in liver metastatic niches of $Kpc1199^{Robo1\text{-FL}}$ modelled CTRL, *Slit2*/CKO or *Slit2*/CKO-RE mice (3 fields assessed per sample, mean±s.e.m.; two tailed unpaired *t*-test). Ki67, red; TUNEL, green; DAPI, blue. Scale bars, 100µm. Source data are provided as Source Data file.

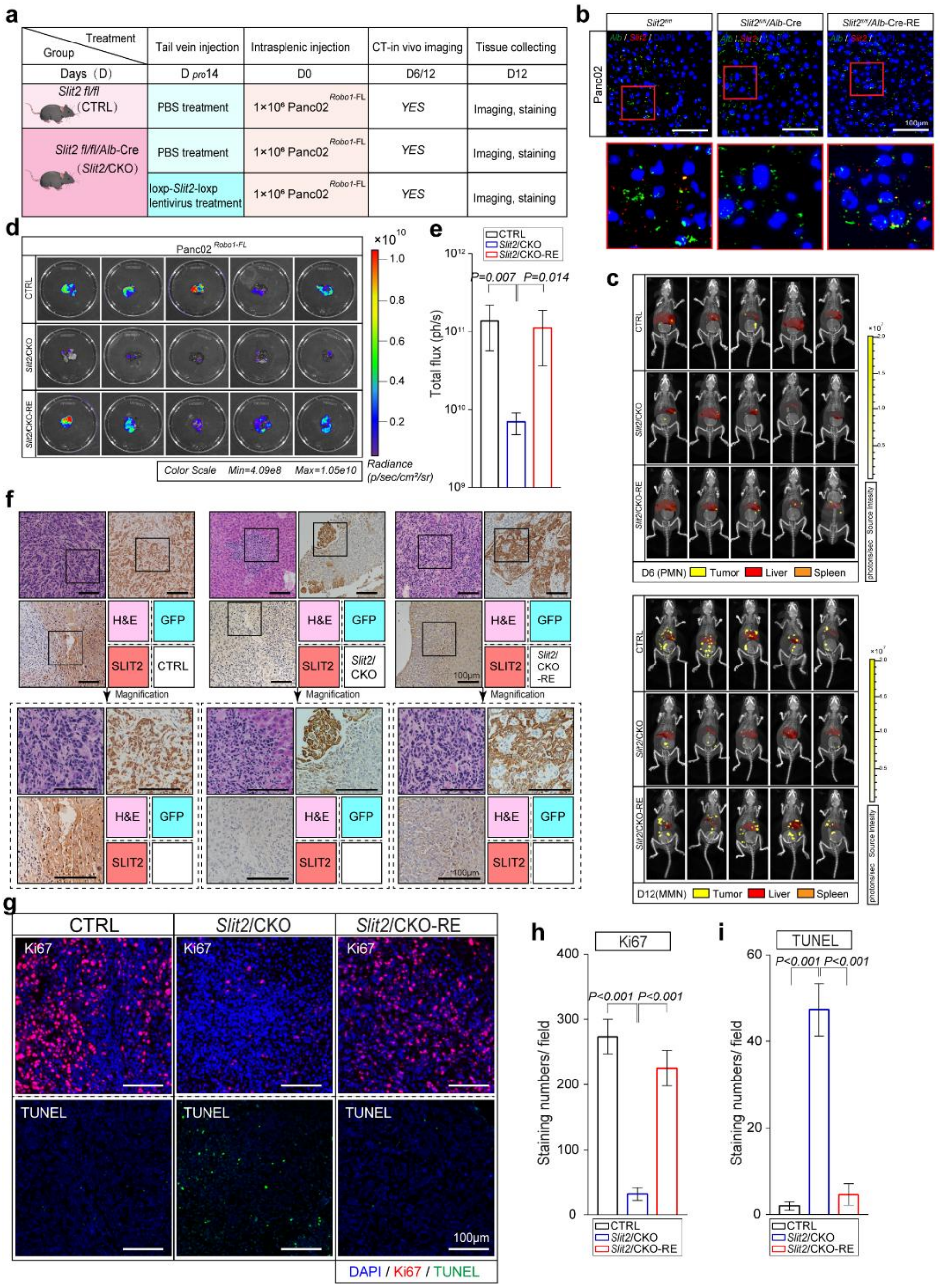


**Supplementary Figure 7. SLIT2 promotes outgrowth of ROBO1$^{+}$ tumour cells at liver metastasis *in vivo* (II)**

(**a**) Brief procedure displaying animal experiment involving CTRL, *Slit2*/CKO or

*Slit2*/CKO-RE mice. (**b**) Representative RNAscope staining examining *Slit2* and *Alb* mRNA in livers of Panc02 modelled $Slit2^{fl/fl}$ or $Slit2^{fl/fl}$/*Alb*-Cre mice with or without Lenti-*loxp*-*Slit2* injection. *Alb*, green; *Slit2*, red; DAPI, blue. (n=5 samples, 3 fields assessed per sample). Scale bar, 100 μm. (**c**) CT combined with 3D organ reconstruction bioluminescence imaging assessing liver metastasis of $Panc02^{Robo1\text{-FL}}$ intrasplenically injected CTRL, *Slit2*/CKO or *Slit2*/CKO-RE mice on PMN (upper) or MMN (lower) (n=5 mice per group). Scale colour bars: $2.00\times10^5$-$2.00\times10^7$. Red, reconstructed liver; orange, reconstructed spleen; yellow, signals of tumour niches. (**d-e**) Assessment of separated metastasized livers from $Panc02^{Robo1\text{-FL}}$ modelled CTRL, *Slit2*/CKO or *Slit2*/CKO-RE mice (n=5 mice per group, mean±s.e.m.; two tailed unpaired *t*-test). Scale colour bar: $4.09\times10^8$-$1.05\times10^{10}$. (**f**) Representative IHC-P staining of H&E, GFP or SLIT2 in left lobule of livers of $Panc02^{Robo1\text{-FL}}$ modelled CTRL, *Slit2*/CKO or *Slit2*/CKO-RE mouse models (n=5 samples per group, 3 fields assessed per sample). Scale bars, 100μm. (**g-i**) Representative staining of Ki67 and TUNEL in liver metastatic niches of $Panc02^{Robo1\text{-FL}}$ modelled CTRL, *Slit2*/CKO or *Slit2*/CKO-RE mice (3 fields assessed per sample, mean±s.e.m.; two tailed unpaired *t*-test). Ki67, red; TUNEL, green; DAPI, blue. Scale bars, 100μm. Source data are provided as Source Data file.

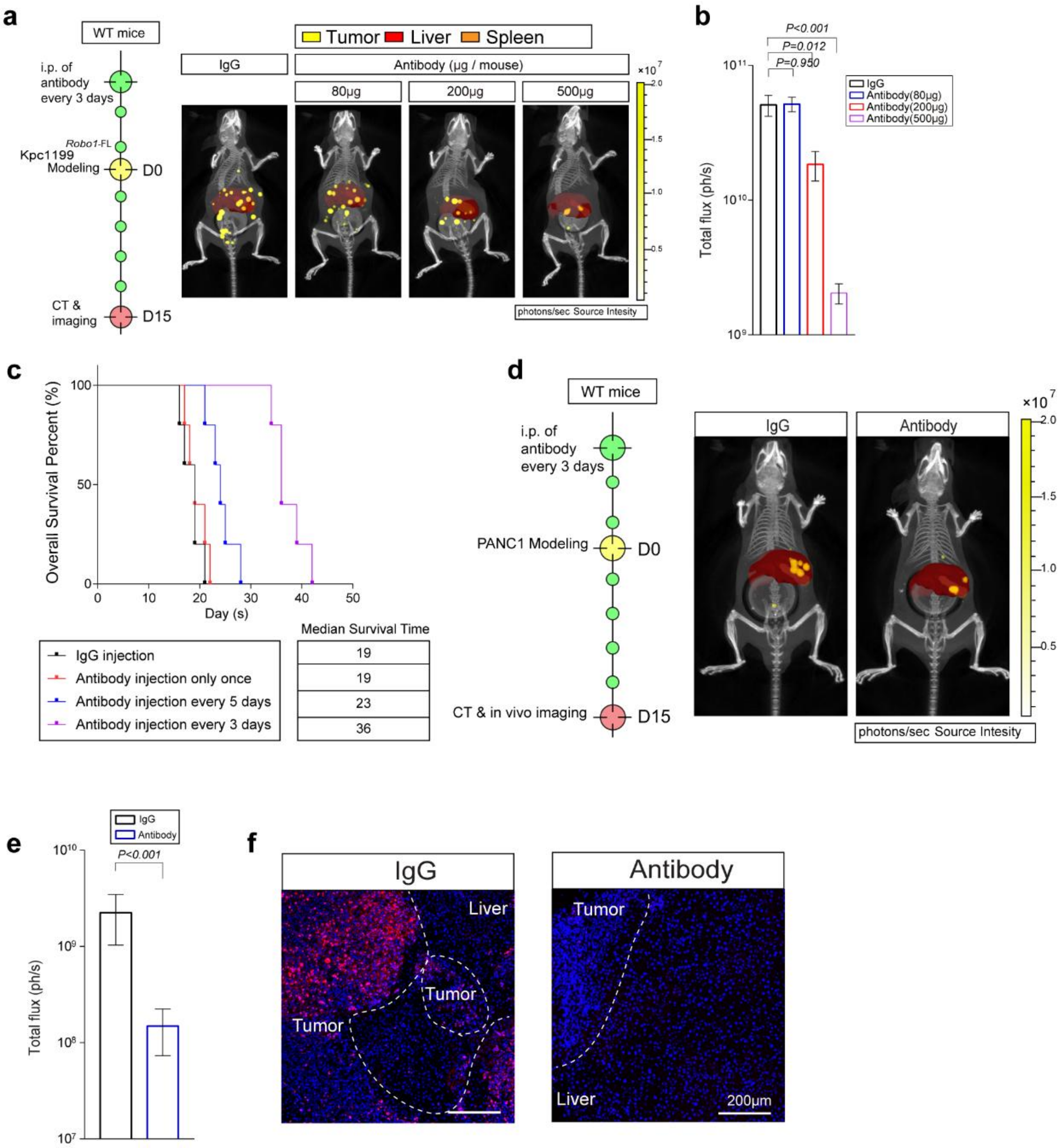


**Supplementary Figure 8. Validation of neutralizing antibody targeting ROBO1**

(**a-b**) Representative CT combined with 3D organ reconstruction bioluminescence imaging displaying Kpc1199$^{Robo1\text{-FL}}$ cell-injected liver metastasis mouse models administered IgG or ROBO1 neutralizing antibody at different concentrations (n=5 mice per group, mean±s.e.m.; two-tailed unpaired *t* test). Scale colour bar:

$2.00\times10^5$-$2.00\times10^7$. Red, reconstructed liver; orange, reconstructed spleen; yellow, signals of tumour niches. (**c**) Survival analysis of intrasplenic mouse models bearing Kpc1199$^{CTRL}$ injection with antibody administration at different strategy (n=5 mice per group). (**d-e**) Representative CT combined with 3D organ reconstruction bioluminescence imaging displaying PANC1 cell-injected liver metastasis mouse models administered IgG or ROBO1 neutralizing antibody. (n=5 mice per group, mean±s.e.m.; two-tailed unpaired *t* test). Scale colour bar: $2.00\times10^5$-$2.00\times10^7$. $P<0.001$. Red, reconstructed liver; orange, reconstructed spleen; yellow, signals of tumour niches. (**f**) Representative staining of Ki67 in liver metastatic niches of PANC1 cell-injected liver metastasis mouse models administered IgG or ROBO1 neutralizing antibody. (3 fields assessed per sample). Ki67, red; DAPI, blue. Scale bars, 200μm. Source data are provided as Source Data file.

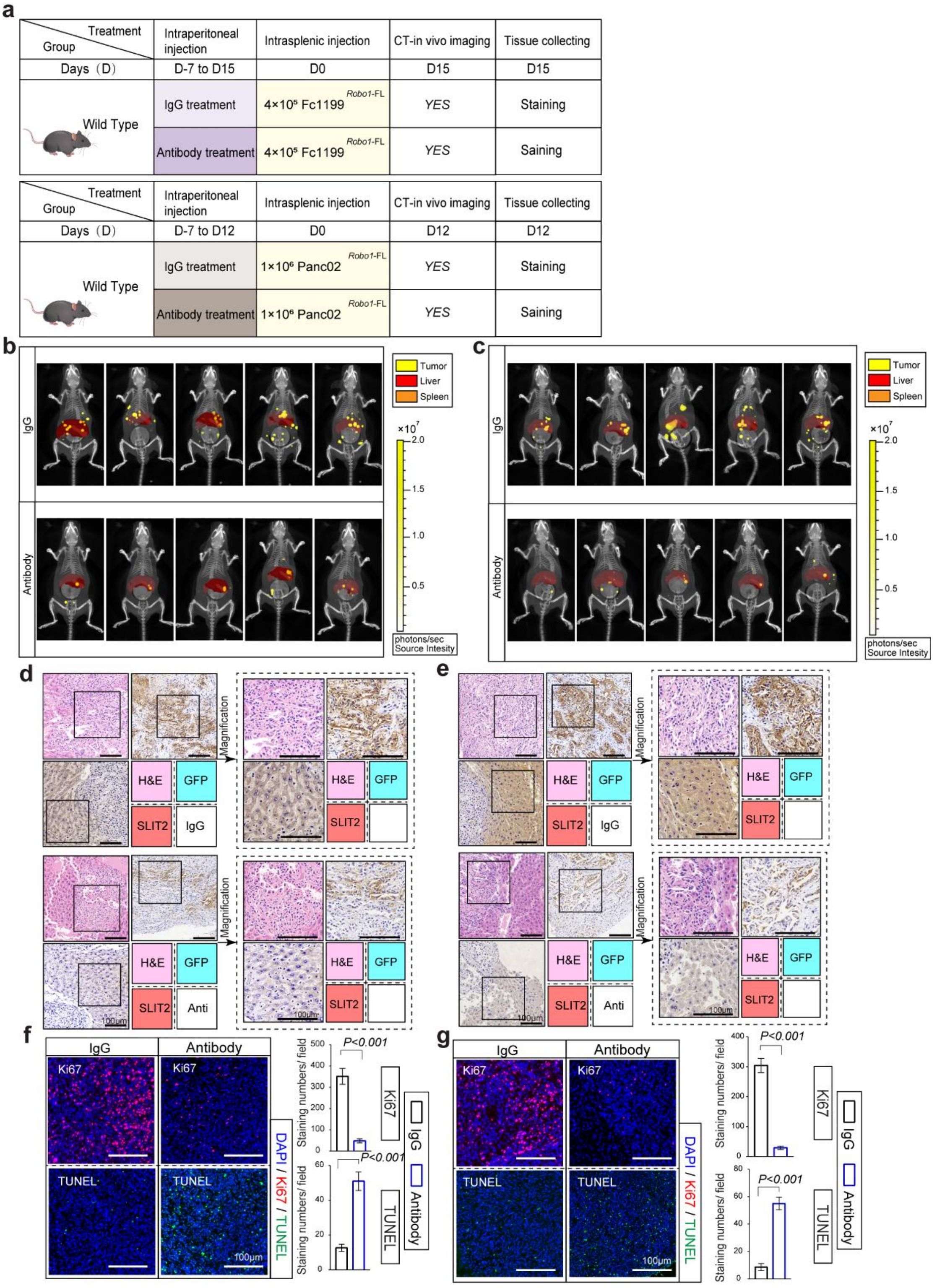


**Supplementary Figure 9. Neutralizing antibody targeting ROBO1 attenuated liver metastasis *in vivo***

(**a**) Brief procedure of liver metastasis mouse model induced by intrasplenic injection of Kpc1199 $^{Robo1}$-FL (upper) or Panc02 $^{Robo1}$-FL (lower) followed by treatment of

anti-ROBO1 neutralizing antibody. (**b-c**) CT combined with 3D organ reconstruction bioluminescence imaging displaying the therapeutic effect of anti-ROBO1 neutralizing antibody in Kpc1199 $^{Robo1\text{-FL}}$ (**b**) or Panc02 $^{Robo1\text{-FL}}$ (**c**) modelled mice. (n=6 mice per group). Scale colour bars: $2.00\times10^5$-$2.00\times10^7$. Red, reconstructed liver; orange, reconstructed spleen; yellow, signals of tumour niches. (**d-e**) Representative IHC-P staining of H&E, GFP or SLIT2 in left lobule of livers of anti-ROBO1 neutralizing antibody-treated Kpc1199$^{Robo1\text{-FL}}$ (**d**) or Panc02 $^{Robo1\text{-FL}}$ (**e**) modelled mice (n=5 samples per group, 3 fields assessed per sample). Scale bars, 100μm. (**f-g**) Representative staining of Ki67 and TUNEL in liver metastatic niches of anti-ROBO1 neutralizing antibody-treated Kpc1199$^{Robo1\text{-FL}}$ (**f**) or Panc02 $^{Robo1\text{-FL}}$ (**g**) modelled mice. (3 fields assessed per sample, mean±s.e.m.; two tailed unpaired *t*-test). Ki67, red; TUNEL, green; DAPI, blue. Scale bars, 100μm. Source data are provided as Source Data file.

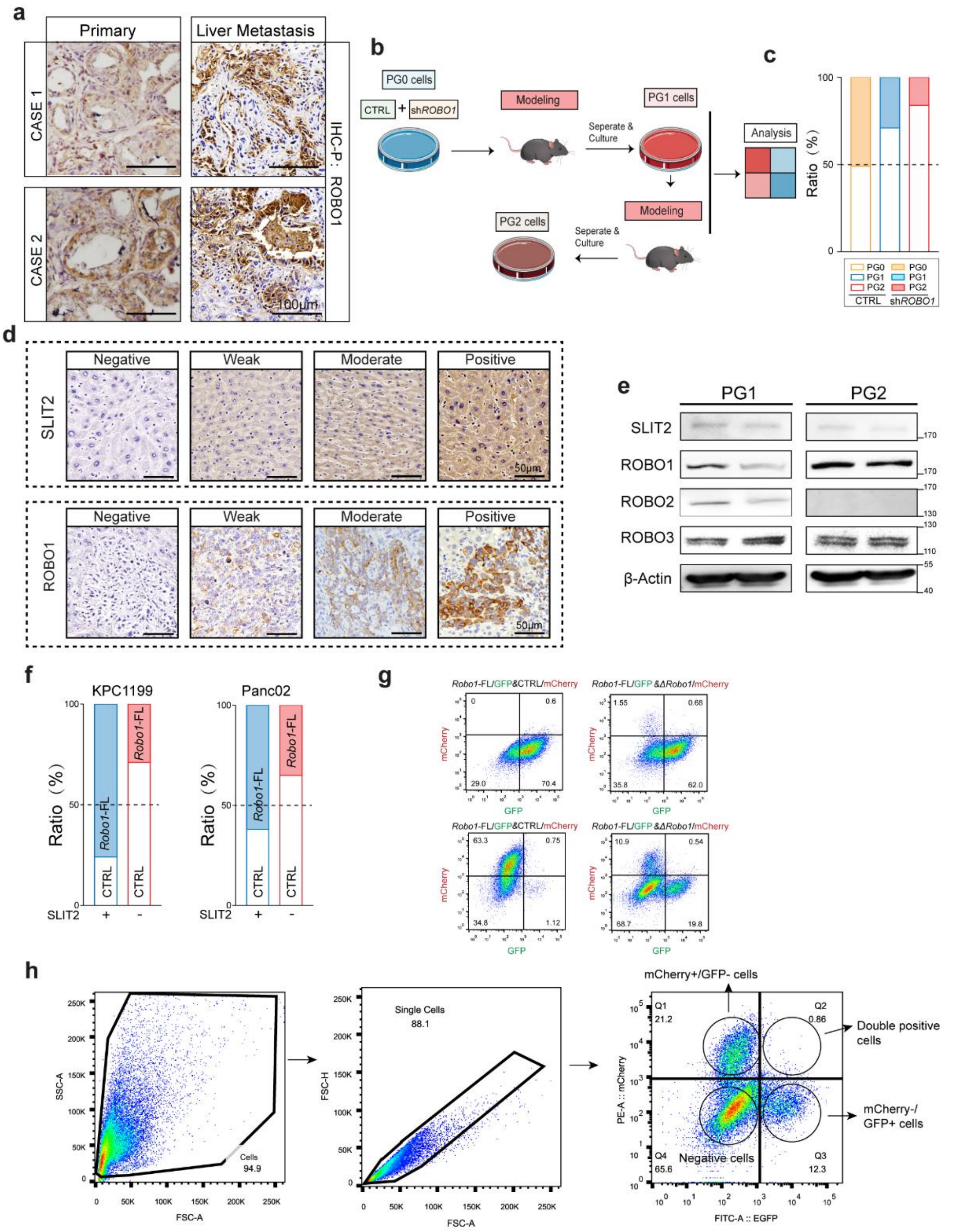


**Supplementary Figure 10. SLIT2-ROBO1 axis generates co-adaption**

(**a**) Representative IHC-P staining of ROBO1 on PDAC primary tumour and paired

liver metastasis of KPC mouse models (n=8 cases, 3 fields assessed per sample). (**b**) Equal amounts of PANC1$^{shCTRL}$ and PANC1$^{Sh\mathit{ROBO1}}$ cells were mixed to prepare an intrasplenic injection model followed by metastatic niche separation and reculture. IHC-P and flow cytometry were performed to evaluate co-adaption. PG0: original cell mixture; PG1: cells derived from PG0-modelled liver metastases; PG2: cells derived from PG1-modelled liver metastases. (**c**) Flow cytometry to detect the cell composition of PG0, PG1 or PG2 (n=5 technical repeats per group). (**d**) Evaluation of SLIT2 or ROBO1 expressions via IHC-P staining on liver metastatic niche of Panc02 modelled mice into four grades: negative, weak, moderate, and positive. (n=32 samples per group, 3 fields assessed per sample). Scale bars, 50μm. (**e**) WB showing the differential expression of SLIT2 or ROBO family in cells of PG1 and PG2(n=3 technical repeats per test). (**f**) Equal amounts of PANC1$^{shCTRL}$ and PANC1$^{Sh\mathit{ROBO1}}$ cells were mixed to seeded in dishes for cell culture with or without SLIT2 treatment. Flow cytometry to detect the cell composition of these two kinds of cells after one generation *in vitro.* (**g**) Flow cytometry detecting the ratio of two kinds of Kpc1199 cells in separated liver metastatic niches. (**h**) Shown is the gating strategy. In brief, all samples were gated to exclude debris and doubles and only living cells were taken into consideration. Cells carrying mCherry or GFP or both were detected using this gating strategy. Source data are provided as Source Data file.

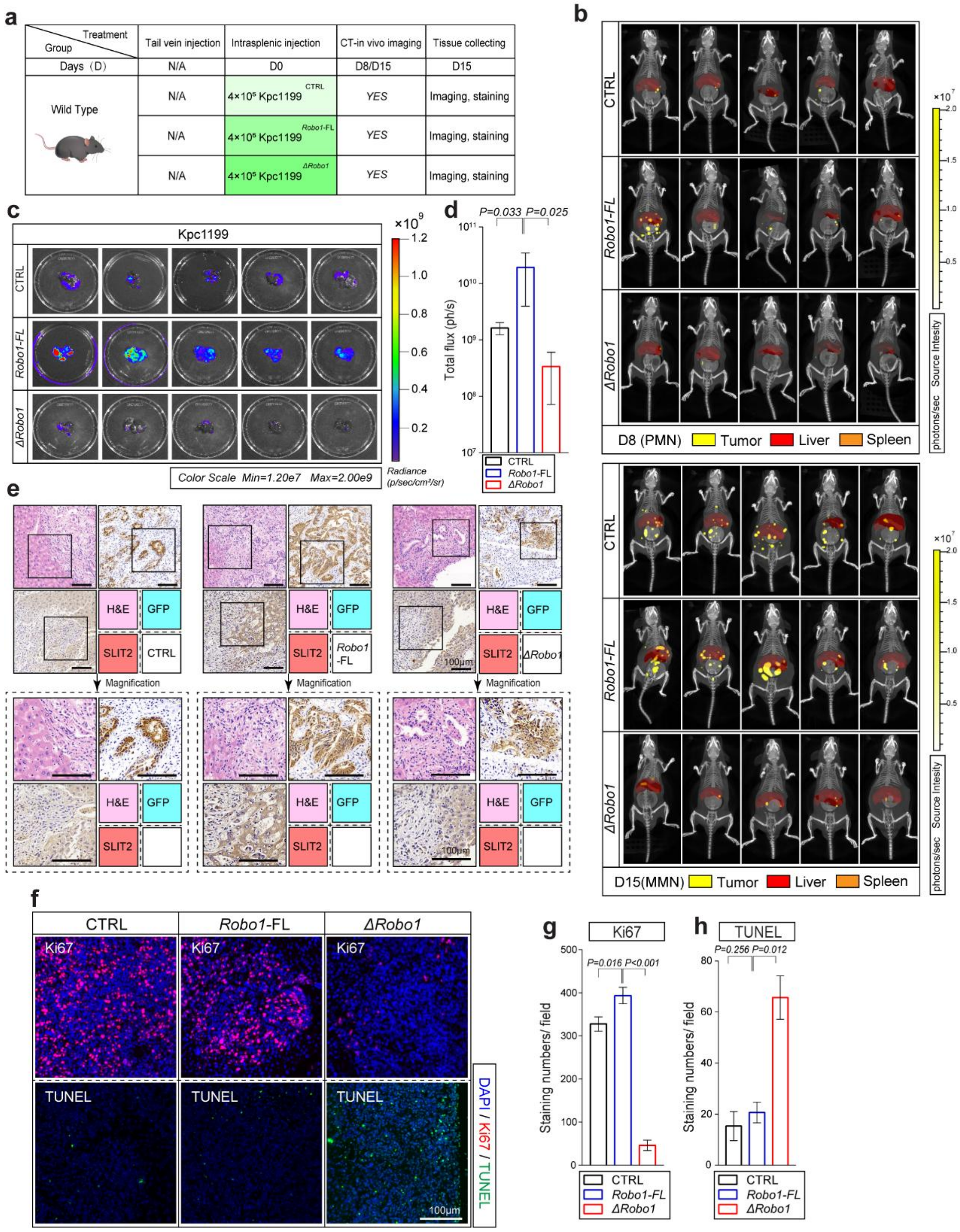


**Supplementary Figure 11. DR ROBO1 modulate co-adaption *in vivo* (I)**

(**a**) Brief procedure of liver metastasis mouse model induced by intrasplenic injection of Kpc1199 expressed *Robo1*-FL or *ΔRobo1*. (**b**) CT combined with 3D organ reconstruction bioluminescence imaging displaying the metastasis ability of Kpc1199$^{\mathrm{CTRL}}$, Kpc1199$^{Robo1\text{-FL}}$ and Kpc1199$^{\Delta Robo1}$ in mouse model on PMN (upper) or

MMN (lower) (n=5 mice per group, mean±s.e.m.; two tailed unpaired *t*-test). Scale colour bars: $2.00\times10^{5}$-$2.00\times10^{7}$. Red, reconstructed liver; orange, reconstructed spleen; yellow, signals of tumour niches. (**c-d**) Assessment of separated metastatic livers from Kpc1199$^{CTRL}$, Kpc1199$^{Robo1\text{-}FL}$ and Kpc1199$^{\Delta Robo1}$ modelled mice (n=5 mice per group, mean±s.e.m.; two tailed unpaired *t*-test). Scale colour bar: $1.20\times10^{7}$-$2.00\times10^{9}$. (**e**) Representative IHC-P staining of H&E, GFP or SLIT2 in left lobule of livers of Kpc1199$^{CTRL}$, Kpc1199$^{Robo1\text{-}FL}$ and Kpc1199$^{\Delta Robo1}$ modelled mice (n=5 samples per group, 3 fields assessed per sample). Scale bars, 100μm. (**f-h**) Representative staining of Ki67 and TUNEL in liver metastatic niches of Kpc1199$^{CTRL}$, Kpc1199$^{Robo1\text{-}FL}$ and Kpc1199$^{\Delta Robo1}$ modelled mice (3 fields assessed per sample, mean±s.e.m.; two tailed unpaired *t*-test). Ki67, red; TUNEL, green; DAPI, blue. Scale bars, 100μm. Source data are provided as Source Data file.

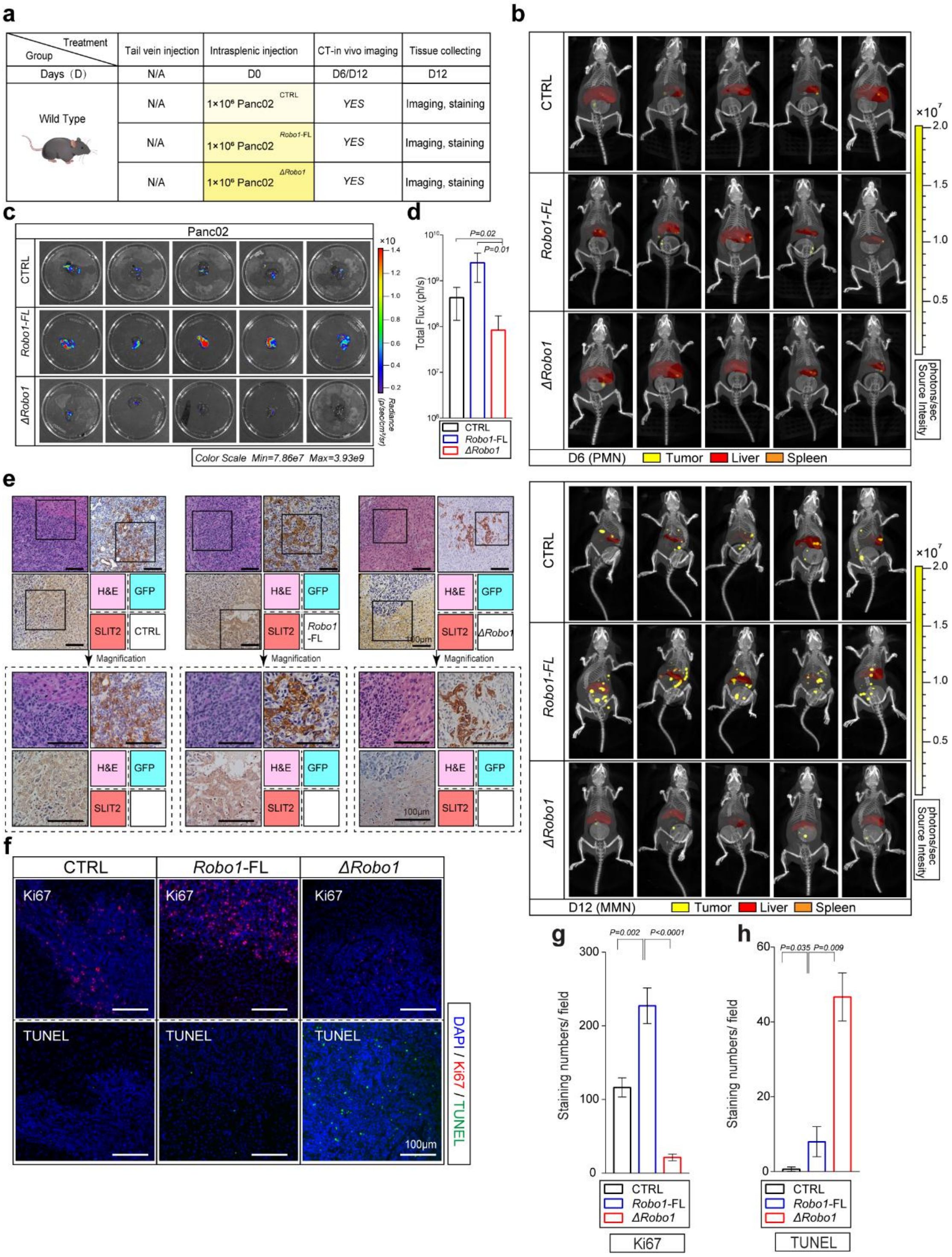


**Supplementary Figure 12. DR ROBO1 modulate co-adaption *in vivo* (II)**

(**a**) Brief procedure of liver metastasis mouse model induced by intrasplenic injection of Panc02 expressed *Robo1*-FL or *ΔRobo1*. (**b**) CT combined with 3D organ reconstruction bioluminescence imaging displaying the metastasis ability of

Panc02$^{CTRL}$, Panc02$^{Robo1\text{-FL}}$ and Panc02$^{\Delta Robo1}$ in mouse models on PMN (upper) or MMN (lower) (n=5 mice per group, mean±s.e.m.; two tailed unpaired *t*-test). Scale colour bars: $2.00\times10^5$-$2.00\times10^7$. Red, reconstructed liver; orange, reconstructed spleen; yellow, signals of tumour niches. (**c-d**) Assessment of separated metastasized livers from Panc02$^{CTRL}$, Panc02$^{Robo1\text{-FL}}$ and Panc02$^{\Delta Robo1}$ modelled mice (n=5 mice per group, mean±s.e.m.; two tailed unpaired *t*-test). Scale colour bar: $7.86\times10^7$-$3.39\times10^9$. (**e**) Representative IHC-P staining of H&E, GFP or SLIT2 in left lobule of livers of Panc02$^{CTRL}$, Panc02$^{Robo1\text{-FL}}$ and Panc02$^{\Delta Robo1}$ modelled mice (n=5 samples per group, 3 fields assessed per sample). Scale bars, 100μm. (**f-h**) Representative staining of Ki67 and TUNEL in liver metastatic niches of Panc02$^{CTRL}$, Panc02$^{Robo1\text{-FL}}$ and Panc02$^{\Delta Robo1}$ modelled mice (3 fields assessed per sample, mean±s.e.m.; two tailed unpaired *t*-test). Ki67, red; TUNEL, green; DAPI, blue. Scale bars, 100μm. Source data are provided as Source Data file.

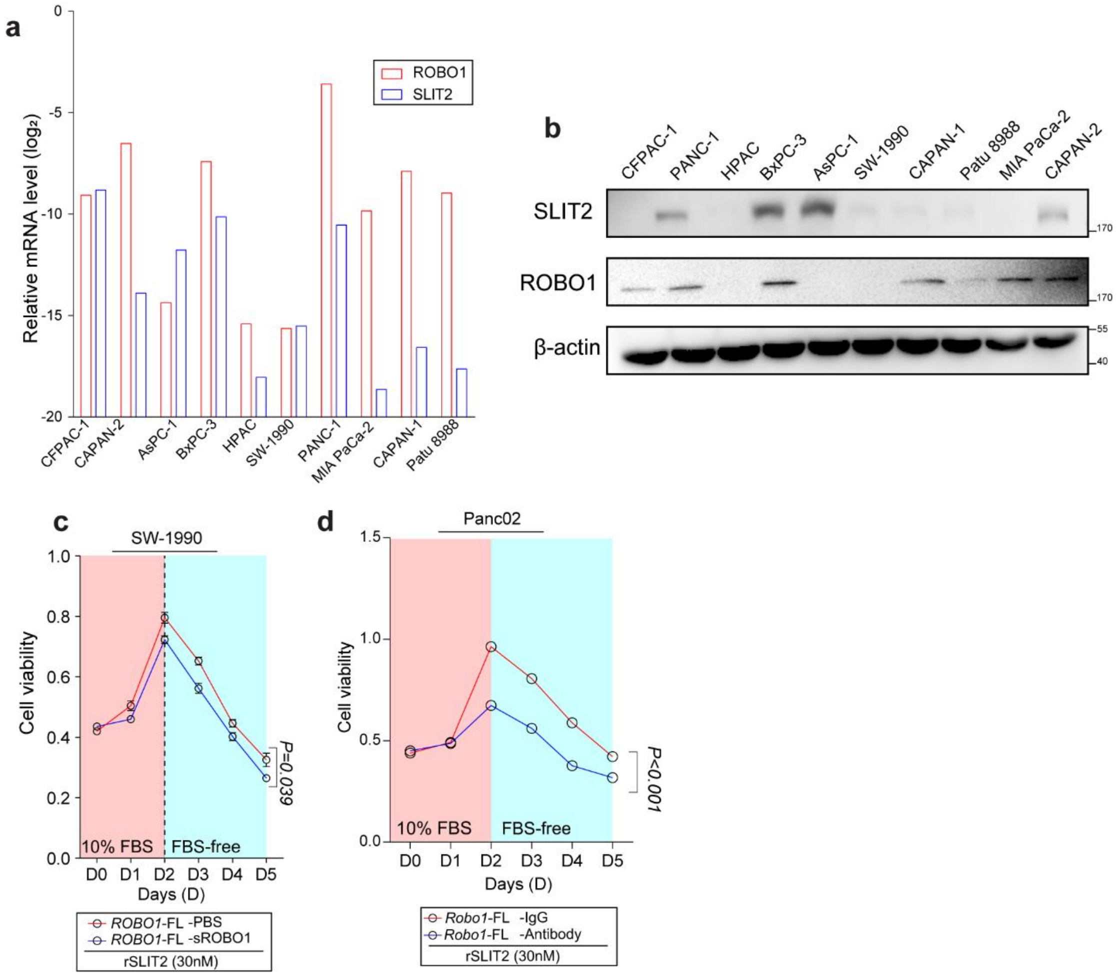


**Supplementary Figure 13. ROBO1 triggers cell growth in vitro (I)**

(**a**) Real-time PCR showing the mRNA levels of SLIT2 and ROBO1 in 10 PDAC cell lines (**b**) WB showing the mRNA levels of SLIT2 and ROBO1 in 10 PDAC cell lines (n=3 technical repeats per test). (**c**) Cell viability of SW-1990 $^{ROBO1\text{-FL}}$ exposed with 30nM rSLIT2 with or without sROBO treatment (n=2 biological replicates, mean±s.e.m. one-way Repeat-Measure ANOVA). Y-axis represented OD values at 450nm. (**d**) Cell viability of Panc02$^{Robo1\text{-FL}}$ exposed with 30nM rSLIT2 with or without anti-ROBO1 neutralizing antibody (n=2 biological replicates, mean±s.e.m. Repeat measures ANOVA). Y-axis represented OD values at 450nm. Source data are provided as Source Data file.

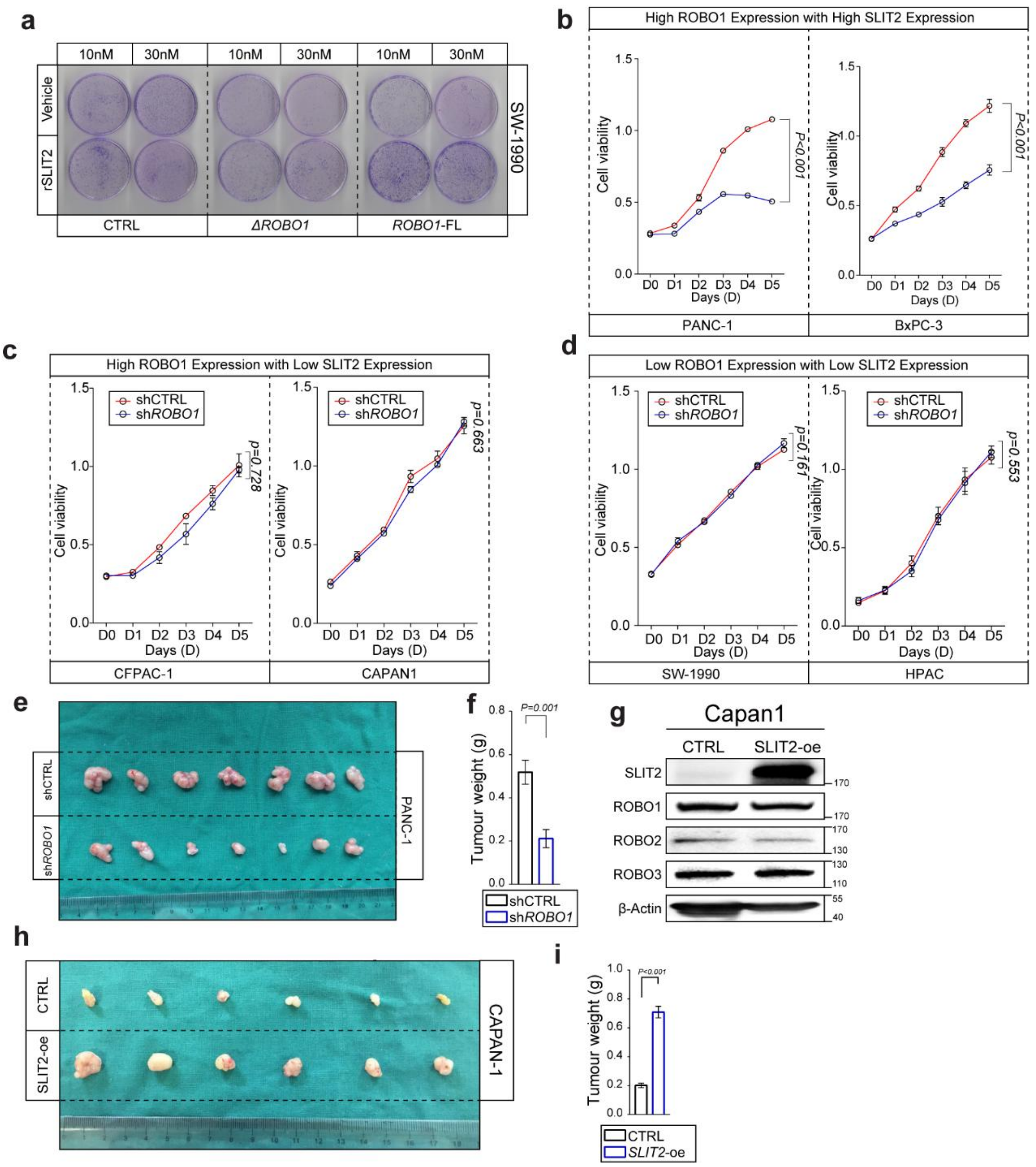


**Supplementary Figure 14. ROBO1 triggers cell growth in vitro (II)**

(**a**) Colony formation assay evaluating outgrowth ability of SW-1990 expressed ROBO1-FL or ΔROBO1 exposed to 10nM or 30nM rSLIT2 (n=2 biological replicates). (**b**) Cell viability of PANC-1 or BxPC-3 cell lines in the presence or absence of shROBO1(n=2 biological replicates, mean±s.e.m. Repeat measures ANOVA). Y-axis represented OD values at 450nm. (**c**) Cell viability of CFPAC-1 or CAPAN1 cell lines in the presence or absence of shROBO1(n=2 biological replicates, mean±s.e.m. Repeat measures ANOVA). *ns.no significant difference, P>0.05.* Y-axis

represented OD values at 450nm. (**d**) Cell viability of SW-1990 or HPAC cell lines in the presence or absence of shROBO1(n=2 biological replicates, mean±s.e.m. Repeat measures ANOVA). *ns.no significant difference, P>0.05.* Y-axis represented OD values at 450nm. (**e**) Subcutaneous xenograft tumours of PANC-1$^{\mathrm{shCTRL}}$ or PANC-1 $^{\mathrm{sh}\mathit{ROBO1}}$ were presented (n=7 mice per group). (**f**) Shown are the tumour weights of subcutaneous xenograft models utilizing PANC-1$^{\mathrm{shCTRL}}$ or PANC-1 $^{\mathrm{sh}\mathit{ROBO1}}$ (n=7 mice per group, mean±s.e.m., two tailed unpaired *t*-test). (**g**) WB assessing the overexpression of SLIT2 in CAPAN-1 cell line (n=3 technical repeats per test). (**h**) Subcutaneous xenograft tumours of CAPAN-1$^{\mathrm{CTRL}}$ or CAPAN-1$^{\mathit{SLIT2}\text{-oe}}$ were presented (n=6 mice per group). (**i**) Shown are the tumour weights of subcutaneous xenograft models utilizing CAPAN-1$^{\mathrm{CTRL}}$ or CAPAN-1$^{\mathit{SLIT2}\text{-oe}}$ (n=6 mice per group, mean±s.e.m.; two tailed unpaired *t*-test). Source data are provided as Source Data file.

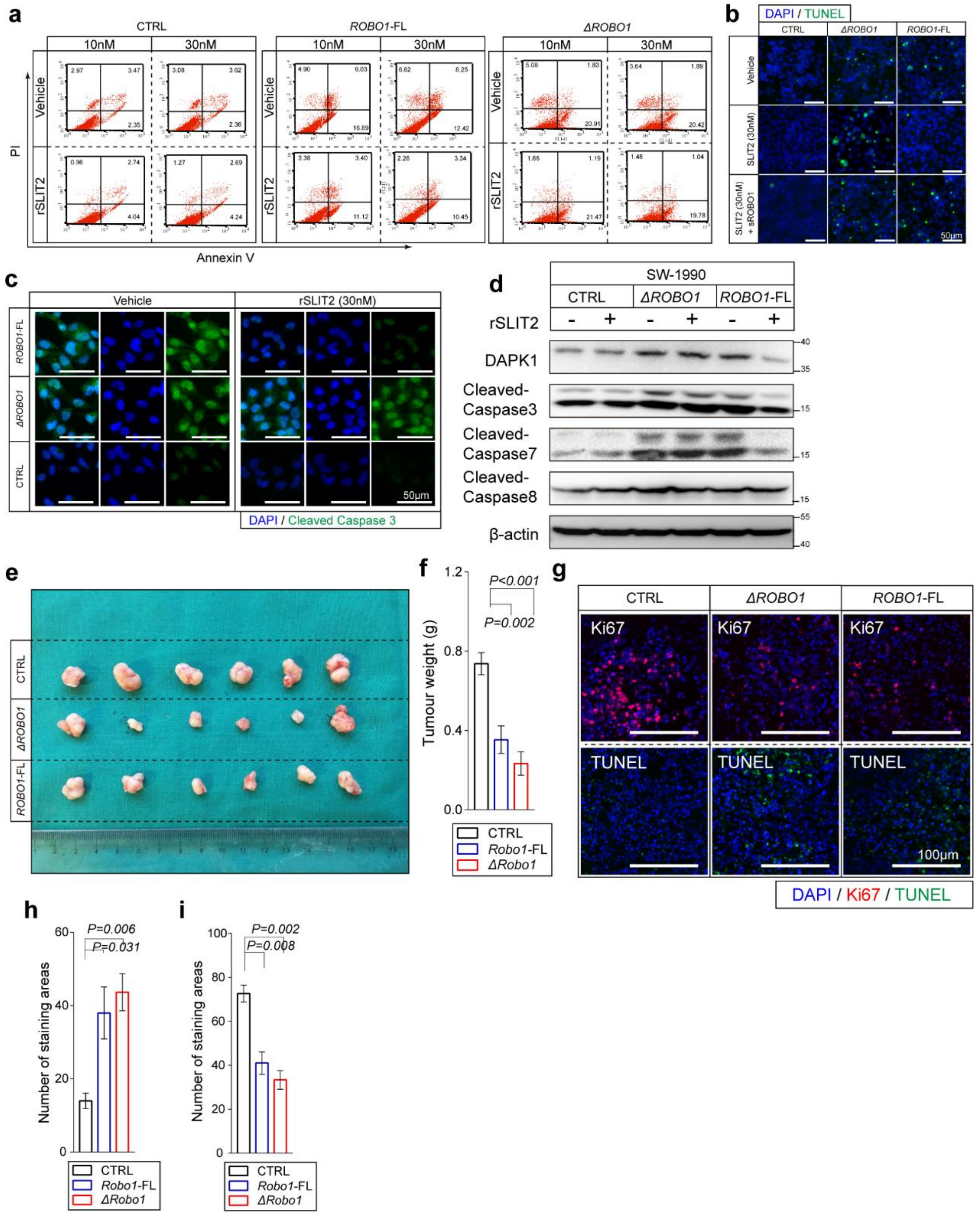

**Supplementary Figure 15. ROBO1 induces cell apoptosis in the absence of SLIT2**
(**a**) Cell apoptosis of SW-1990$^{CTRL}$, SW-1990$^{\Delta ROBO1}$ and SW-1990$^{ROBO1\text{-FL}}$ with or without rSLIT2 administration (10nM or 30nM) measured by flow cytometry with dual staining of PI and Annexin V (n=2 biological replicates, n=3 tests per group). (**b**)

TUNEL assay performed on SW-1990$^{\mathrm{CTRL}}$, SW-1990$^{\Delta ROBO1}$ and SW-1990$^{ROBO1\text{-FL}}$ with or without 30nM rSLIT2 exposure; for blocking, sROBO administration is performed to SW-1990$^{ROBO1\text{-FL}}$ with rSLIT2 exposure (n=3 biological replicates, 3 fields assessed per sample). TUNEL staining, green; DAPI, blue. Scale bars: 50μm. (**c**) IF displaying the level of activated caspase 3 in SW-1990$^{\mathrm{CTRL}}$, SW-1990$^{\Delta ROBO1}$ and SW-1990$^{ROBO1\text{-FL}}$ with or without 30nM rSLIT2 administration (3 fields assessed per sample). Caspase 3, green; DAPI, blue. Scale bars: 50μm. (**d**) WB evaluating the caspase pathway triggered by unbound ROBO1. (**e**) Subcutaneous xenograft tumours of SW-1990$^{\mathrm{CTRL}}$, SW-1990$^{\Delta ROBO1}$ and SW-1990$^{ROBO1\text{-FL}}$ were presented (n=6 mice per group). (**f**) Shown are the tumour weights of subcutaneous xenograft models utilizing SW-1990$^{\mathrm{CTRL}}$, SW-1990$^{\Delta ROBO1}$ and SW-1990$^{ROBO1\text{-FL}}$ at day 24 (n=6 mice per group, mean±s.e.m., two tailed unpaired *t*-test). (**g-i**) Representative IHC-P staining of Ki67 and TUNEL performed on subcutaneous xenograft tumours of SW-1990$^{\mathrm{CTRL}}$, SW-1990$^{\Delta ROBO1}$ and SW-1990$^{ROBO1\text{-FL}}$ (3 fields assessed per sample; mean±s.e.m.; two tailed unpaired *t*-test). Ki67, red; TUNEL, green. Scale bars: 100μm. Source data are provided as Source Data file.

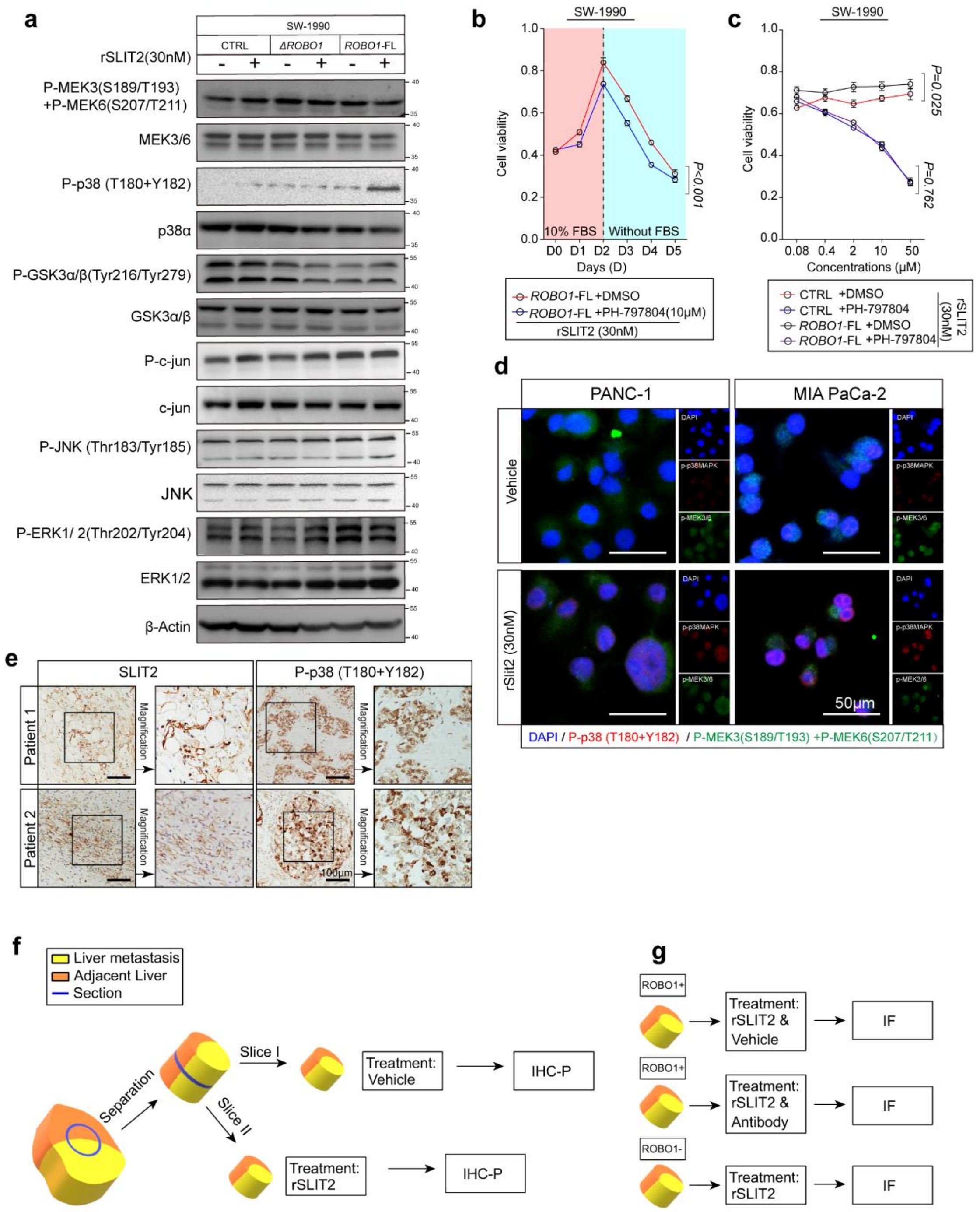

**Supplementary Figure 16. SLIT2-ROBO1 axis triggers p38MAPK pathway activation**

(**a**) WB assessing the activation of MAPK pathway in SW-1990$^{CTRL}$, SW-1990$^{\Delta ROBO1}$

and SW-1990$^{ROBO1\text{-FL}}$ at exposure of 30nM rSLIT2 (n=3 technical repeats per test). (**b**) Cell viability of SW-1990$^{ROBO1\text{-FL}}$ exposed with 30nM rSLIT2 with or without p38α specific inhibitor PH-797804 (50μM) (n=5 technical repeats per group, mean±s.e.m. one-way Repeat-Measure ANOVA). Y-axis represented OD values at 450nm. (**c**) Cell viability of SW-1990$^{\text{CTRL}}$ and SW-1990$^{ROBO1\text{-FL}}$ with 30nM rSLIT2 with or without p38α specific inhibitor PH-797804 at various concentrations (0.08μM, 0.4μM 2μM, 10μM, 50μM; Time point=D3; n=3 technical repeats per group, mean±s.e.m. one-way Repeat-Measure ANOVA). Y-axis represented OD values at 450nm. (**d**) Representative IF staining in 30nM rSLIT2 treated PANC-1 and MIA PaCa-2 (3 fields assessed per sample). P-p38 (T180+Y182), red; P-MEK3 (S189/T193) +P-MEK6 (S207/T211), green; DAPI, blue. Scale bars: 50μm. (**e**) Representative IHC-P staining of SLIT2 (left) or P-p38 (T180+Y182) (right) (n=4 samples, 3 fields assessed per sample). Scale bar, 100μm. (**f-g**) Brief schematic diagram on procedure of living tissue culture of liver metastasis separated from intrasplenic model (**f**) or KPC mice (**g**). Source data are provided as Source Data file.

| ID | AGE | GENDER |
|---|---|---|
| 1 | 60 | Female |
| 2 | 55 | Male |
| 3 | 71 | Male |
| 4 | 82 | Female |
| 5 | 40 | Male |
| 6 | 50 | Female |
| 7 | 68 | Female |
| 8 | 58 | Male |
| 9 | 58 | Male |
| 10 | 63 | Male |
| 11 | 65 | Male |
| 12 | 69 | Male |
| 13 | 69 | Female |
| 14 | 75 | Male |
| 15 | 47 | Male |
| 16 | 69 | Male |
| 17 | 69 | Female |
| 18 | 64 | Male |
| 19 | 58 | Male |
| 20 | 58 | Female |
| 21 | 53 | Male |
| 22 | 61 | Male |
| 23 | 77 | Male |
| 24 | 59 | Female |
| 25 | 62 | Female |
| 26 | 68 | Male |
| 27 | 58 | Female |
| 28 | 67 | Female |
| 29 | 59 | Male |
| 30 | 53 | Female |
| 31 | 57 | Male |
| 32 | 63 | Male |
| 33 | 68 | Male |
| 34 | 66 | Female |
| 35 | 73 | Male |

**Supplementary Table 1.** Cohort I: 35 cases PDAC patients bearing liver metastasis

| ID | AGE | GENDER |
|---|---|---|
| 1 | 73 | Male |
| 2 | 70 | Male |
| 3 | 52 | Female |
| 4 | 64 | Male |
| 5 | 74 | Female |
| 6 | 69 | Male |
| 7 | 78 | Female |
| 8 | 74 | Female |
| 9 | 63 | Male |
| 10 | 62 | Male |
| 11 | 70 | Female |
| 12 | 69 | Female |
| 13 | 81 | Male |
| 14 | 50 | Male |
| 15 | 84 | Male |
| 16 | 47 | Male |
| 17 | 59 | Male |
| 18 | 55 | Male |
| 19 | 69 | Male |
| 20 | 52 | Male |
| 21 | 80 | Female |
| 22 | 83 | Female |
| 23 | 76 | Male |
| 24 | 55 | Female |
| 25 | 38 | Female |
| 26 | 61 | Male |
| 27 | 65 | Male |
| 28 | 73 | Male |
| 29 | 58 | Male |
| 30 | 65 | Male |
| 31 | 73 | Male |
| 32 | 54 | Male |
| 33 | 56 | Male |
| 34 | 52 | Male |
| 35 | 72 | Male |
| 36 | 76 | Male |
| 37 | 60 | Male |
| 38 | 64 | Male |
| 39 | 74 | Male |

| | | |
|---|---|---|
| 40 | 43 | Female |
| 41 | 57 | Male |
| 42 | 75 | Male |
| 43 | 57 | Male |
| 44 | 80 | Male |
| 45 | 66 | Male |
| 46 | 64 | Female |
| 47 | 69 | Male |
| 48 | 70 | Female |
| 49 | 61 | Female |
| 50 | 41 | Female |
| 51 | 61 | Male |
| 52 | 62 | Female |
| 53 | 85 | Female |
| 54 | 64 | Male |
| 55 | 50 | Male |
| 56 | 80 | Female |
| 57 | 60 | Female |
| 58 | 74 | Female |
| 59 | 74 | Female |
| 60 | 41 | Male |
| 61 | 78 | Female |
| 62 | 63 | Female |
| 63 | 71 | Female |
| 64 | 83 | Female |
| 65 | 58 | Male |
| 66 | 53 | Female |
| 67 | 80 | Female |
| 68 | 74 | Female |
| 69 | 57 | Male |
| 70 | 55 | Male |
| 71 | 80 | Female |
| 72 | 78 | Female |
| 73 | 63 | Female |
| 74 | 72 | Male |
| 75 | 71 | Male |
| 76 | 63 | Male |
| 77 | 70 | Female |
| 78 | 69 | Female |
| 79 | 65 | Male |
| 80 | 77 | Male |
| 81 | 58 | Female |
| 82 | 57 | Female |

| | | |
|---|---|---|
| 83 | 73 | Female |
| 84 | 77 | Female |
| 85 | 55 | Male |
| 86 | 60 | Male |
| 87 | 76 | Female |
| 88 | 68 | Female |
| 89 | 50 | Male |
| 90 | 60 | Male |
| 91 | 68 | Female |
| 92 | 68 | Male |
| 93 | 58 | Male |
| 94 | 79 | Male |
| 95 | 68 | Female |
| 96 | 69 | Female |
| 97 | 78 | Female |
| 98 | 57 | Female |
| 99 | 60 | Female |
| 100 | 68 | Male |
| 101 | 57 | Male |
| 102 | 53 | Male |
| 103 | 71 | Male |
| 104 | 75 | Female |
| 105 | 71 | Female |
| 106 | 74 | Male |
| 107 | 66 | Male |
| 108 | 69 | Female |
| 109 | 59 | Male |
| 110 | 76 | Female |
| 111 | 66 | Male |
| 112 | 72 | Female |
| 113 | 65 | Male |
| 114 | 40 | Male |
| 115 | 40 | Female |
| 116 | 56 | Male |
| 117 | 79 | Female |
| 118 | 65 | Male |
| 119 | 41 | Male |
| 120 | 60 | Male |
| 121 | 76 | Male |
| 122 | 69 | Female |
| 123 | 40 | Male |
| 124 | 78 | Male |
| 125 | 72 | Male |

| | | |
|---|---|---|
| 126 | 64 | Male |
| 127 | 72 | Female |
| 128 | 85 | Male |
| 129 | 59 | Male |
| 130 | 73 | Female |
| 131 | 57 | Male |
| 132 | 74 | Female |
| 133 | 67 | Male |
| 134 | 58 | Male |
| 135 | 44 | Male |
| 136 | 59 | Female |
| 137 | 71 | Male |
| 138 | 56 | Female |
| 139 | 77 | Female |
| 140 | 85 | Female |
| 141 | 83 | Male |
| 142 | 56 | Female |
| 143 | 54 | Male |
| 144 | 70 | Female |
| 145 | 79 | Female |
| 146 | 38 | Male |
| 147 | 76 | Male |
| 148 | 57 | Female |
| 149 | 63 | Male |
| 150 | 59 | Male |
| 151 | 55 | Male |
| 152 | 62 | Female |
| 153 | 69 | Female |
| 154 | 58 | Female |
| 155 | 77 | Male |
| 156 | 62 | Male |
| 157 | 68 | Female |
| 158 | 59 | Female |
| 159 | 75 | Female |
| 160 | 59 | Male |
| 161 | 52 | Male |
| 162 | 55 | Female |
| 163 | 56 | Female |
| 164 | 68 | Female |
| 165 | 43 | Male |
| 166 | 80 | Male |
| 167 | 62 | Male |
| 168 | 57 | Female |

| | | |
|---|---|---|
| 169 | 60 | Male |
| 170 | 65 | Female |
| 171 | 65 | Female |
| 172 | 53 | Male |
| 173 | 90 | Female |
| 174 | 63 | Female |
| 175 | 72 | Male |
| 176 | 60 | Female |
| 177 | 52 | Female |
| 178 | 60 | Male |
| 179 | 65 | Male |
| 180 | 82 | Male |
| 181 | 55 | Male |
| 182 | 54 | Male |
| 183 | 81 | Male |
| 184 | 63 | Male |
| 185 | 65 | Female |
| 186 | 57 | Male |
| 187 | 58 | Female |
| 188 | 81 | Male |
| 189 | 85 | Female |
| 190 | 75 | Female |
| 191 | 89 | Male |
| 192 | 54 | Male |
| 193 | 53 | Female |
| 194 | 79 | Female |
| 195 | 66 | Male |
| 196 | 46 | Male |
| 197 | 78 | Male |
| 198 | 60 | Female |
| 199 | 60 | Male |
| 200 | 65 | Male |
| 201 | 54 | Male |
| 202 | 67 | Male |
| 203 | 76 | Female |
| 204 | 71 | Male |
| 205 | 71 | Male |
| 206 | 58 | Female |
| 207 | 77 | Female |
| 208 | 91 | Female |
| 209 | 49 | Male |
| 210 | 71 | Male |
| 211 | 63 | Male |

| | | |
|---|---|---|
| 212 | 74 | Female |
| 213 | 57 | Female |
| 214 | 42 | Male |
| 215 | 63 | Female |
| 216 | 74 | Female |
| 217 | 47 | Female |
| 218 | 70 | Female |
| 219 | 79 | Male |
| 220 | 57 | Male |
| 221 | 66 | Male |
| 222 | 72 | Male |
| 223 | 57 | Male |
| 224 | 77 | Female |
| 225 | 84 | Male |
| 226 | 70 | Male |
| 227 | 73 | Male |
| 228 | 62 | Male |
| 229 | 51 | Male |
| 230 | 83 | Male |
| 231 | 73 | Female |
| 232 | 57 | Male |
| 233 | 60 | Female |
| 234 | 71 | Female |
| 235 | 67 | Male |
| 236 | 71 | Male |
| 237 | 60 | Female |
| 238 | 37 | Male |
| 239 | 81 | Male |
| 240 | 63 | Male |
| 241 | 66 | Male |
| 242 | 58 | Male |
| 243 | 65 | Male |
| 244 | 75 | Female |
| 245 | 67 | Female |
| 246 | 74 | Male |
| 247 | 69 | Male |
| 248 | 69 | Female |
| 249 | 61 | Male |
| 250 | 61 | Male |
| 251 | 53 | Male |
| 252 | 70 | Female |
| 253 | 71 | Male |
| 254 | 66 | Female |

| | | |
|---|---|---|
| 255 | 63 | Male |
| 256 | 63 | Female |
| 257 | 78 | Male |
| 258 | 50 | Female |
| 259 | 73 | Female |
| 260 | 69 | Male |
| 261 | 67 | Male |
| 262 | 89 | Male |
| 263 | 67 | Male |
| 264 | 70 | Female |
| 265 | 57 | Female |
| 266 | 70 | Female |

**Supplementary Table 2.** Cohort III: 266 cases PDAC patients
