# Supplementary material for: Coadaptation fostered by the SLIT2-ROBO1 axis facilitates liver metastasis of pancreatic ductal adenocarcinoma
Source: Nat Commun. 2023 Feb 15;14:861. doi: 10.1038/s41467-023-36521-0 (PMC9932171; doi:10.1038/s41467-023-36521-0)
Supplement: Supplementary file 3 — Reporting Summary [file 41467_2023_36521_MOESM3_ESM.pdf]

nature portfolio

Corresponding author(s): Zhi-Gang Zhang, Jun Li, Yong-Wei Sun

Last updated by author(s): Feb 3, 2023

Reporting Summary

Nature Portfolio wishes to improve the reproducibility of the work that we publish. This form provides structure for consistency and transparency in reporting. For further information on Nature Portfolio policies, see our Editorial Policies and the Editorial Policy Checklist.

Statistics

For all statistical analyses, confirm that the following items are present in the figure legend, table legend, main text, or Methods section.

n/a	Confirmed	
☐	☒	The exact sample size (n) for each experimental group/condition, given as a discrete number and unit of measurement
☐	☒	A statement on whether measurements were taken from distinct samples or whether the same sample was measured repeatedly
☐	☒	The statistical test(s) used AND whether they are one- or two-sided<br>*Only common tests should be described solely by name; describe more complex techniques in the Methods section.*
☒	☐	A description of all covariates tested
☐	☒	A description of any assumptions or corrections, such as tests of normality and adjustment for multiple comparisons
☐	☒	A full description of the statistical parameters including central tendency (e.g. means) or other basic estimates (e.g. regression coefficient) AND variation (e.g. standard deviation) or associated estimates of uncertainty (e.g. confidence intervals)
☐	☒	For null hypothesis testing, the test statistic (e.g. F, t, r) with confidence intervals, effect sizes, degrees of freedom and P value noted<br>*Give P values as exact values whenever suitable.*
☒	☐	For Bayesian analysis, information on the choice of priors and Markov chain Monte Carlo settings
☒	☐	For hierarchical and complex designs, identification of the appropriate level for tests and full reporting of outcomes
☒	☐	Estimates of effect sizes (e.g. Cohen's d, Pearson's r), indicating how they were calculated

Our web collection on statistics for biologists contains articles on many of the points above.

Software and code

Policy information about availability of computer code

Data collection	ChemiDocTM XRS+ system (BIO-RAD) and Odyssey imaging system were used for WB imaging; Flow cytometry signals were detected in BD Fortessa FACS with FACSDiva software v6.0. CT combined 3D Organ Reconstruction Bioluminescence Imaging was performed on Caliper Life Sciences; IF was observed on Leica TCS SP8 LAS X; CCK-8 assay was measured on Tecan Infinite microplate reader system. In vivo imaging data were collected by IVIS Spectrum version 4.5.3.
Data analysis	We utilized Software IBM SPSS statistics 19.0 and GraphPad 7.0 for normal data analysis. Flow cytometry data was analyzed using FlowJo Software version 10.4.2 software. Gene set enrichment analysis were performed by 4GB (64bit) GSEA v4.01 Java Web Start (all platforms). In vivo imaging were assessed by IVIS Spectrum version 4.5.3.

For manuscripts utilizing custom algorithms or software that are central to the research but not yet described in published literature, software must be made available to editors and reviewers. We strongly encourage code deposition in a community repository (e.g. GitHub). See the Nature Portfolio guidelines for submitting code & software for further information.

Data

Policy information about availability of data

All manuscripts must include a data availability statement. This statement should provide the following information, where applicable:

- Accession codes, unique identifiers, or web links for publicly available datasets
- A description of any restrictions on data availability
- For clinical datasets or third party data, please ensure that the statement adheres to our policy

All data generated or analyzed involved in this study are provided or cited in our article.
For RNA-seq on mice liver metastasis the analysis data is available in the Sequence Read Archive (SRA), the number of which is PRJNA590588. This database contains 2 parts of analysis data: Panc02 cell line modelled liver metastasis part (Panc02_D3_(1-3),Panc02_D6_(1-3) and Panc02_D12_(1-3)) and KPC1199 cell line modelled liver metastasis part (D3_(1-3),D8_(1-3) and D15_(1-3)).
For ROBO1 and SLIT2 expression analysis, the datasets is GSE (https://www.ncbi.nlm.nih.gov/geo/query/acc.cgi?acc=GSE71729);
For EMT pathway analysis, the datasets are GEODATASET GSE15471(https://www.ncbi.nlm.nih.gov/geo/query/acc.cgi?acc=GSE15471) and https://portal.gdc.cancer.gov/projects/TCGA-PAAD.

Human research participants

Policy information about studies involving human research participants and Sex and Gender in Research.

Reporting on sex and gender	These information could be available on Extended Data Table1-Table2
Population characteristics	The detailed participants characteristics such as age and gender could be available on Extended Data Table1-Table2
Recruitment	All cases of PDAC or PDAC liver metastases were obtained from Ren Ji hospital from February 2004 to September 2014. All patients had not received radiotherapy, chemotherapy, hormone therapy or other related anti-tumor therapies before surgery.
Ethics oversight	The study was approved by the Research Ethics Committee of Ren Ji Hospital, School of Medicine, Shanghai Jiao Tong University. Written informed consent was provided before enrollment.Approval letter of Shanghai Jiaotong University School of Medicine, Renji Hospital Ethics Committee is RA-2019-116.1.

Note that full information on the approval of the study protocol must also be provided in the manuscript.

Field-specific reporting

Please select the one below that is the best fit for your research. If you are not sure, read the appropriate sections before making your selection.

☒ Life sciences ☐ Behavioural & social sciences ☐ Ecological, evolutionary & environmental sciences

For a reference copy of the document with all sections, see nature.com/documents/nr-reporting-summary-flat.pdf

Life sciences study design

All studies must disclose on these points even when the disclosure is negative.

Sample size	For samples from patients: specimens involved in this study mainly included 2 cohorts for respective experimental designs: Cohort I containing 35 cases liver metastasis tissues of PDAC patients together with paired primary tumor tissues was used for IHC-P or IF analysis; cohort II containing 266 cases PDAC primary tumor tissue of patients with their respective prognosis was performed for IHC-P and survival analysis. Overall,the sample sizes of PDAC patients cohorts were determined by the number of tumor samples and clinic information available in our database.<br>For mice experiments, 5-7 mice were used per group. Sample size and number of independent experiments are always clearly stated in the figure legend or in the Methods section and are repeated three or more times independent for statistical analyses.<br>All samples were not predetermined with statistical methods, but based on expected effect size. And the cost and feasibility were also taken into consideration.
Data exclusions	None
Replication	Experiments in the article are reliably produced, replication were described in the figure legends.
Randomization	All mice and cells were randomly assigned to experimental groups.

Blinding	Investigators are blinded to group allocation.

Reporting for specific materials, systems and methods

We require information from authors about some types of materials, experimental systems and methods used in many studies. Here, indicate whether each material, system or method listed is relevant to your study. If you are not sure if a list item applies to your research, read the appropriate section before selecting a response.

Materials & experimental systems

n/a	Involved in the study
☐	☒ Antibodies
☐	☒ Eukaryotic cell lines
☒	☐ Palaeontology and archaeology
☐	☒ Animals and other organisms
☐	☒ Clinical data
☐	☒ Dual use research of concern

Methods

n/a	Involved in the study
☒	☐ ChIP-seq
☐	☒ Flow cytometry
☒	☐ MRI-based neuroimaging

Antibodies

Antibodies used

Antibodies against the following proteins were purchased from Abcam: ROBO1 (C-terminal) (ab7279)(IP,1:100;WB,1:1000), SLIT2 (ab134166)(WB,1:1000;IHC-P,1:500), ROBO2 (ab75014)(WB,1:1000;IHC-P,1:300), ROBO3 (ab229722)(WB,1:500;IHC-P,1:500), TIMP1 (ab109125)(IHC-P,1:500), LOX (ab174316)(IHC-P,1:500), MIF (ab7027)(IHC-P,1:300), SAA1/2 (ab199030)(IHC-P,1:700), CD163 (ab182422)(IHC-P,1:300), CD68 (ab955)(IHC-P,1:100), CD11b (ab133357)(IHC-P,1:200), Ly-6G (ab25377)(IHC-P,1:300), CK19 (rabbit-derived) (ab52625)(IHC-P,1:500), CK19 (mouse-derived)(ab7754)(IHC-P,1:500), N-cadherin (ab76011)(IHC-P,1:500), E-cadherin (ab1416)(IHC-P,1:500), RAC1 + Cdc42 (phospho S71) (ab76535)(IHC-P,1:500), P-p38 (T180+Y182) (ab4822)(WB,1:1000;IHC-P,1:500), P-MEK3 (S189/T193)+P-MEK6 (S207/T211) (ab4759)(WB,1:500), MEK3+MEK6 (ab200831)(IP,1:50;WB,1:1000), active Caspase 3 (ab2302), albumin (ab207327)(1:500),beta-actin(ab8226)(WB,1:10000),donkey anti-goat IgG H&L (HRP) (ab6885)(WB,1:5000;IHC-P,1:1000), and rabbit IgG (ab172730)(using at a concentration according to respective primary antibody),Alexa Fluor® 680-conjugated anti-rabbit (ab175773)(WB,1:15000), Alexa Fluor® 790-conjugated anti-mouse (ab175783)(WB,1:15000), Goat Anti-Rabbit IgG H&L (HRP) (ab6721)(WB,1:1000;IHC-P,1:500) and Goat Anti-Mouse IgG H&L (HRP)(ab6789)(WB,1:1000;IHC-P,1:500).
Antibodies against the following proteins were purchased from Cell Signaling Technology: DAPK1 (3008)(WB,1:1000), active Caspase 7 (9491)(WB,1:1000), active Caspase 8 (9748)(WB,1:1000), JNK (9252)(WB,1:1000), P-JNK (Thr183/Tyr185) (9251)(WB,1:1000), ERK1/2 (4695)(WB,1:1000), P-ERK1/2 (T202/Y204) (4370)(WB,1:1000), c-jun (9165)(WB,1:1000), P-c-jun (9261)(WB,1:1000), GSK3α/β (5676)(WB,1:1000), P-GSK3α/β (Y216/Y279)(9327)(WB,1:1000), and mouse IgG (37988)(using at a concentration according to respective primary antibody).
P38α MAPK antibody (orb540329)(IP,1:100;WB,1:1000;IHC-P,1:700), was purchased from Biorbyt.
A neutralizing antibody against the Ig1-Ig2 domain of ROBO1 (HAM1H6-1-8) and negative control IgG were purchased from HuaBio (IP,1:100;WB,1:500;IHC-P,1:300).

Validation

All the antibodies involved in this study were selected and utilized based on manufactures' validations. Antibody validation information can be found on manufacturers' website:
ROBO1 (C-terminal) (ab7279):https://www.abcam.cn/robo1-antibody-ab7279.html;SLIT2 (ab134166):https://www.abcam.cn/slit2-antibody-epr2771-ab134166.html;ROBO2 (ab75014):https://www.abcam.cn/robo2--robo3-antibody-ab75014.html;ROBO3 (ab229722):https://www.abcam.cn/robo3-antibody-ab229722.html;TIMP1 (ab109125):https://www.abcam.cn/timp1-antibody-epr1550-ab109125.html;LOX (ab174316):https://www.abcam.cn/lox-antibody-epr4025-ab174316.html;MIF (ab7027):https://www.abcam.cn/prmt1-antibody-mat-b12-ab7027.html;SAA1/2 (ab199030):https://www.abcam.cn/saa1--saa2-antibody-epr19235-ab199030.html;CD163 (ab182422):https://www.abcam.cn/cd163-antibody-epr19518-ab182422.html;CD68 (ab955):https://www.abcam.cn/cd68-antibody-kp1-ab955.html;CD11b (ab133357):https://www.abcam.cn/cd11b-antibody-epr1344-ab133357.html;Ly-6G (ab25377):https://www.abcam.cn/ly6g--ly6c-antibody-rb6-8c5-ab25377.html;CK19 (rabbit-derived) (ab52625):https://www.abcam.cn/cytokeratin-19-antibody-ep1580y-cytoskeleton-marker-ab52625.html;CK19 (mouse-derived)(ab7754):https://www.abcam.cn/cytokeratin-19-antibody-a53-ba2-cytoskeleton-marker-ab7754.html;N-cadherin (ab76011):https://www.abcam.cn/n-cadherin-antibody-epr1791-4-ab76011.html;E-cadherin (ab1416):https://www.abcam.cn/e-cadherin-antibody-hecd-1-intercellular-junction-marker-ab1416.html;RAC1 + Cdc42 (phospho S71) (ab76535):https://www.abcam.cn/rac1--cdc42-phospho-s71-antibody-ep221y-ab76535.html;P-p38 (T180+Y182) (ab4822):https://www.abcam.cn/p38-phospho-t180--y182-antibody-ab4822.html;P-MEK3 (S189/T193)+P-MEK6 (S207/T211) (ab4759):https://www.abcam.cn/products?keywords=P-MEK3+%28S189%2FT193%29%2BP-MEK6+%28S207%2FT211%29+%28ab4759%29;MEK3+MEK6 (ab200831):https://www.abcam.cn/mek3--mek6-antibody-epr17340-ab200831.html;active Caspase 3 (ab2302):https://www.abcam.cn/cleaved-caspase-3-antibody-ab2302.html;albumin (ab207327):https://www.abcam.cn/albumin-antibody-epr20195-ab207327.html;beta-actin(ab8226):https://www.abcam.cn/beta-actin-antibody-mabcam-8226-loading-control-ab8226.html;DAPK1 (3008):https://www.cellsignal.cn/products/primary-antibodies/dapk1-antibody/3008;active Caspase 7 (9491):https://www.cellsignal.cn/products/primary-antibodies/cleaved-caspase-7-asp198-antibody/9491?site-search-type=Products&N=4294956287&Ntt=9491&fromPage=plp&_requestid=9315665;active Caspase 8 (9748):https://www.cellsignal.cn/products/primary-antibodies/cleaved-caspase-8-asp384-11g10-mouse-mab/9748?site-search-type=Products&N=4294956287&Ntt=9748&fromPage=plp&_requestid=9315959;JNK (9252):https://www.cellsignal.cn/products/primary-antibodies/sapk-jnk-antibody/9252?site-search-type=Products&N=4294956287&Ntt=9252&fromPage=plp&_requestid=9316584;P-JNK (Thr183/Tyr185) (9251):https://www.cellsignal.cn/products/primary-antibodies/phospho-sapk-jnk-thr183-tyr185-antibody/9251?site-search-type=Products&N=4294956287&Ntt=9251&fromPage=plp&_requestid=9316927;ERK1/2 (4695):https://www.cellsignal.cn/products/

primary-antibodies/p44-42-mapk-erk1-2-137f5-rabbit-mab/4695?site-search-type=Products&N=4294956287&Ntt=4695&fromPage=plp;P-ERK1/2 (T202/Y204) (4370):https://www.cellsignal.cn/products/primary-antibodies/phospho-p44-42-mapk-erk1-2-thr202-tyr204-d13-14-4e-xp-rabbit-mab/4370?site-search-type=Products&N=4294956287&Ntt=4370&fromPage=plp;c-jun (9165):https://www.cellsignal.cn/products/primary-antibodies/c-jun-60a8-rabbit-mab/9165?site-search-type=Products&N=4294956287&Ntt=9165&fromPage=plp&_requestid=9328568;P-c-jun (9261):https://www.cellsignal.cn/products/primary-antibodies/phospho-c-jun-ser63-ii-antibody/9261?site-search-type=Products&N=4294956287&Ntt=9261&fromPage=plp&_requestid=9306294;GSK3α/β (5676):https://www.cellsignal.cn/products/primary-antibodies/gsk-3a-b-d75d3-rabbit-mab/5676?site-search-type=Products&N=4294956287&Ntt=5676&fromPage=plp&_requestid=9306583;P-GSK3α/β (Y216/Y279)(9327):https://www.cellsignal.cn/products/primary-antibodies/phospho-gsk-3a-b-ser21-9-37f11-rabbit-mab-gsk-3a-preferred/9327?site-search-type=Products&N=4294956287&Ntt=gsk3%CE%B1%2F%CE%B2&fromPage=plp;P38α MAPK antibody (orb540329):https://www.biorbyt.com/mapk14-antibody-orb540329.html
Neutralizing antibody against the Ig1-Ig2 domain of ROBO1 (HAM1H6-1-8) was customized, and the validation of which could be available on Extended Data Figure 8. Validation of neutralizing antibody targeting ROBO1.

Eukaryotic cell lines

Policy information about cell lines and Sex and Gender in Research

Cell line source(s)	Human PDAC cell lines PANC-1, BxPC-3, CFPAC-1, HPAC, CAPAN-1, CAPAN-2, Patu 8988, MIA PaCa-2, SW-1990 and AsPC-1 were purchased from ATCC; Murine cell lines Panc02, Kpc1199 and LTPA were gifting from Professor Jing Xue (State Key Laboratory of Oncogenes andRelated Genes, Shanghai Cancer Institute, Ren Ji Hospital, School of Medicine, Shanghai Jiao Tong University).
Authentication	All cell lines were validated using short tandem repeat (STR) profiling.
Mycoplasma contamination	All cell lines involved in this study were without mycoplasma contamination.
Commonly misidentified lines (See ICLAC register)	None

Animals and other research organisms

Policy information about studies involving animals; ARRIVE guidelines recommended for reporting animal research, and Sex and Gender in Research

Laboratory animals	lox-stop-lox-KrasG12D/+; lox-stop-lox-Trp53R172H/+; Pdx1-Cre (KPC)mice were purchased from The Jackson Laboratories (Bar Harbor, ME) to generate transgenic PDAC mouse model. Slit2fl/fl mice and Alb1-Cre mice were purchased from Cyagen. 16-24 weeks old KPC mice and 8 weeks old WT or Slit2 KO mice or nu/nu mice were performed in this study. All mice above were on the C57BL/6 genetic background.<br>Animals were housed in East China Normal University SPF animal facility, in temperatures 20-22℃, humidity 30-70% and a 12-hour light/12-hour dark cycle.
Wild animals	none
Reporting on sex	Equal ratio of male and female
Field-collected samples	This study did not involve field-collected samples
Ethics oversight	Animal experiments were approved by Institutional Animal Care and Use Committee of East China Normal University.All manipulations were performed under approved protocol number 20141204 assigned by the Research Ethics Committee of East China Normal University.

Note that full information on the approval of the study protocol must also be provided in the manuscript.

Clinical data

Policy information about clinical studies
All manuscripts should comply with the ICMJE guidelines for publication of clinical research and a completed CONSORT checklist must be included with all submissions.

Clinical trial registration	No clinical trial.
Study protocol	Study protocols can be seen in detail in this article
Data collection	Data collection can be seen in detail in this article
Outcomes	None

Dual use research of concern

Policy information about dual use research of concern

Hazards

Could the accidental, deliberate or reckless misuse of agents or technologies generated in the work, or the application of information presented in the manuscript, pose a threat to:

No	Yes	
☒	☐	Public health
☒	☐	National security
☒	☐	Crops and/or livestock
☒	☐	Ecosystems
☒	☐	Any other significant area

Experiments of concern

Does the work involve any of these experiments of concern:

No	Yes	
☒	☐	Demonstrate how to render a vaccine ineffective
☒	☐	Confer resistance to therapeutically useful antibiotics or antiviral agents
☒	☐	Enhance the virulence of a pathogen or render a nonpathogen virulent
☒	☐	Increase transmissibility of a pathogen
☒	☐	Alter the host range of a pathogen
☒	☐	Enable evasion of diagnostic/detection modalities
☒	☐	Enable the weaponization of a biological agent or toxin
☒	☐	Any other potentially harmful combination of experiments and agents

Flow Cytometry

Plots

Confirm that:

☒ The axis labels state the marker and fluorochrome used (e.g. CD4-FITC).

☒ The axis scales are clearly visible. Include numbers along axes only for bottom left plot of group (a 'group' is an analysis of identical markers).

☒ All plots are contour plots with outliers or pseudocolor plots.

☒ A numerical value for number of cells or percentage (with statistics) is provided.

Methodology

Sample preparation	For apoptosis analysis measured by Annexin V & propidium iodide (PI) staining: Cells were detached with 0.25% trypsin/0.01% EDTA in 1×PBS after treatment with or without rSLIT2 administration (10nM or 30nM). Then suspended cells were harvested in DMEM and centrifuged at 800rpm for 3 minutes. These cells were then stained with 3.5µl Annexin V and 3.5µl PI diluted in 100µl binding buffer after 1×PBS washing.<br>For animal experiments, the protocol could be available in "Metastatic Tumor Dissociation, Culture and Examination" in this paper. In brief, liver metastatic tumors were washed by icy DMEM for 3 times and cut into 1×1×1mm cubes before transferred into 2.5 mL DMEM containing 100 µL of Enzyme D, 50 µL of Enzyme R, and 12.5 µL of Enzyme A provided in kit. After suspending the dissociated tumor tissues in the solution mentioned before, the tubes were tightly closed and put on constant temperature oscillator for 1h at 37℃, 120 rpm. The mixtures were then performed centrifugation at 1000 rpm for 5 min and the samples would be resuspended in DMEM for 3 times before examination.
Instrument	BD Fortessa FACS
Software	FACSDiva software v6.0.
Cell population abundance	In apoptosis measurement, cell population abundance was pre-estimated using cell counting chamber to confirm consistency; for animal experiments, tumor cell purity (tumor cells / all cells)was greater than 95%.
Gating strategy	Gating strategy can be clearly gained in Extended Data Figure 10h

☒ Tick this box to confirm that a figure exemplifying the gating strategy is provided in the Supplementary Information.
